# First report of *trans*-A_2_B-corrole derived from a lapachone derivative: photophysical, TD-DFT and photobiological assays†

**DOI:** 10.1039/d3ra00823a

**Published:** 2023-04-11

**Authors:** Bruna M. Rodrigues, Carlos C. Diniz, Vinicius N. da Rocha, Mateus H. Köhler, Guilherme P. Brandão, Luana A. Machado, Eufranio N. da Silva Júnior, Bernardo A. Iglesias

**Affiliations:** a Bioinorganic and Porphyrinoid Material Laboratory, Department of Chemistry, Federal University of Santa Maria Santa Maria-RS Brazil bernardo.iglesias@ufsm.br; b Department of Physics, Federal University of Santa Maria Santa Maria-RS Brazil; c Department of Chemistry, Federal University of Minas Gerais Belo Horizonte MG Brazil; d Julius Maximilians-Universität Würzburg (JMU), Institute for Inorganic Chemistry Am Hubland Würzburg 97074 Germany

## Abstract

In this work, the synthesis, characterization and photophysical assays of a new *trans*-A_2_B-corrole derivative from the naturally occurring quinone are described. β-Lapachone is a naturally occurring quinoidal compound that provides highly fluorescent heterocyclic compounds such as lapimidazoles. The new *trans*-A_2_B-corrole compound was obtained from the reaction between 2,3,4,5,6-(pentafluorophenyl)dipyrromethane and the lapimidazole bearing an aldehyde group. The dyad was characterized by high-resolution mass spectrometry (HRMS), NMR spectroscopy (^1^H, COSY 2D, HMBC, ^19^F), FT-IR, UV-vis, steady-state and time-resolved fluorescence, electrochemical studies (CV), TD-DFT analysis and photobiological experiments, in which includes aggregation, stability in solution, photostability and partition coefficients assays. Finally, ROS generation assays were performed using 1,3-diphenylisobenzofuran (DPBF) method and the presented compound showed significant photostability and singlet oxygen production.

## Abstract 2

Photophysical properties of novel corroles containing lapachol units. DFT calculations and electrochemical analysis were conducted to better understand the corrole molecular structures. Corrole derivatives do not form aggregates in solution and generate ROS species.

## Graphical abstract

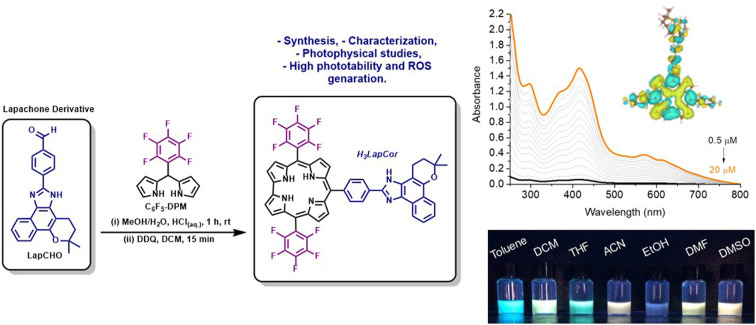

## 1. Introduction

Fluorescent compounds are indispensable tools for a wide range of applications for several purposes such as fluorescent sensors,^1^ OLEDs,^2^ and bioimaging studies.^3^

**Scheme 1:**
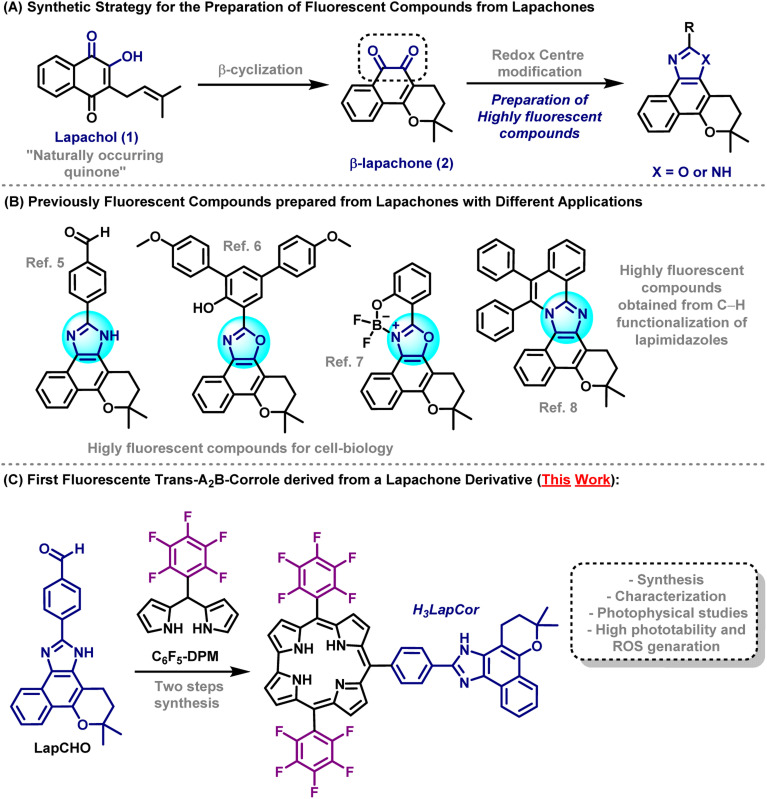
Overview.

Lapachol (1) is a naturally occurring quinoidal compound isolated from the heartwood of trees from the Bignoniaceae family, known in Latin America as the Ipe tree and possesses a wide range of biological activities.^4^ Lapachol moiety is the synthetic precursor of β-lapachone (2), in which the redox centre modification^4*c*,*d*^ allows the synthesis of outstanding heterocyclic compounds featuring prominent fluorescent properties such as lapimidazoles and lapoxazoles (Scheme 1A).

In recent years, our research group has made efforts to construct novel fluorescent compounds based on lapachone moiety for various applications such as selective fluorescent mitochondrial imaging probes,^5^ lapoxalozes derivatives for imaging of lipid droplets^6^ and, fluorescent boron-complexed lapoxazoles as superior and selective probes for endocytic pathway tracking in live cancer cells.^7^ Recently, our research group also reported the synthesis of highly fluorescent compounds based on lapachone derivatives through transition metal catalysed C–H annulation reactions. These protocols provided a one-step fashion to achieve highly extended compounds with remarkable photophysical properties^8^ (Scheme 1B).

Corroles are tetrapyrrole macrocycles in the class of porphyrins commonly used as sensitizers in photodynamic processes, including photodynamic therapy (PDT) and antimicrobial photodynamic therapy (aPDT). Depending on the electronic structure (substituents, metal ion center) these derivatives may present good photostability, high reactive oxygen species (ROS) production yield, and interaction with target biomacromolecules.^9,10^ A number of charges, aggregation state, and hydrophobicity character are determining factors for the compounds to have a promising photodynamic action.^9,10^ The study of ROS formation is fundamental for photodynamic assays, requiring the combination of a photosensitizer (PS), molecular oxygen (O_2_), and the adequate light source. In this case, there are two main reaction mechanisms: type I, which produces radical species (˙OH, O_2_˙^−^, ˙OOH) by electron/hole transfer, and type II, which might generate ^1^O_2_ species by energy transfer processes.^11^ Due to their high reactivity, ROS can oxidize different biological substrates and biomacromolecules, including nucleic acids, lipoproteins, serum albumin, microorganisms, and cells.^12^

In the present manuscript, we report the synthesis of new mono-substituted corrole containing lapachone unit at *meso* position (H_3_LapCor) (Scheme 1C). Their characterization by high-resolution mass spectrometry, FT-IR and NMR techniques were recorded. The photophysical study by absorption, steady-state and time-resolved fluorescence emission analysis was conducted in several solvents, with TD-DFT calculations analysis. In addition, photobiological properties, including aggregation, stability, photostability, coefficient partition values, and ROS were conducted and studied.

## 2 Experimental

### 2.1. General

All chemical reagents were of analytical grade and purchased from Sigma-Aldrich® and Oakwood Chemical® (USA) without any further purification. Column chromatography was carried out using silica-flash 230–400 mesh from Aldrich. Analytical preparative thin-layer chromatography was performed on aluminum sheets (1.0 mm thick) Merck TLC silica-gel 60.

Compounds were analyzed using a high-resolution mass spectrometer with electrospray ionization (HRMS-ESI) in the positive mode using a micrOTOF-QII mass spectrometer (Bruker Daltonics, Billerica, MA). Mass spectrum was recorded for each sample in methanolic solution (concentration of 500 ppb) with flow of 4.0 μL min^−1^ and capillarity of 3500 V. ^1^H, ^19^F, COSY 2D and HMBC NMR spectra were recorded with a Bruker Avance III spectrometer at 600 (^1^H) and 565 MHz (^19^F) respectively, using deuterated chloroform (CDCl_3_) as the solvent and tetramethylsilane (TMS) as the internal reference. The chemical shifts are expressed in *δ* (ppm) and coupling constants (*J*) are given in Hertz (Hz). The multiplicities are expressed as: s, singlet; d, duplet, and m, multiplet. Fourier-transform infrared spectroscopy (FT-IR) spectra was recorded on a Bruker Vertex 70 spectrometer equipped with diamond attenuated total reflectance (ATR) accessory in the 4000–400 cm^−1^ region.

### 2.2. Synthesis of corrole H_3_LapCor

The compounds aldehyde LapCHO and 2,3,4,5,6-(pentafluorophenyl)dipyrromethane C_6_F_5_-DPM were synthesized according to the literature.^5,13^ Dipyrromethane C_6_F_5_-DPM (0.159 g; 0.512 mmol) and the aldehyde LapCHO (0.091 g; 0.256 mmol) were dissolved in 50 mL MeOH. Subsequently, a solution of HCl_aq_ (37%; 2.5 mL) in H_2_O (50 mL) was added and the reaction was stirred at room temperature for 1 h. In the next step, the mixture was extracted with DCM, and the organic layer was washed twice with H_2_O, dried (with anhydrous Na_2_SO_4_), filtered, and diluted to 250 mL with DCM. A solution of DDQ (0.115 g; 0.512 mmol) in toluene/dichloromethane mixture (1 : 3 v/v; 8 mL) was added, and the mixture was stirred for 15 min. The reaction mixture was concentrated to half of this volume, and it was purified first on a silica-gel chromatography column (ethyl acetate/*n*-hexane, 60 : 40). Then, the resulting residue was recrystallized using a mixture of DCM/*n*-hexane (20 : 80) to obtain pure dark greenish corrole H_3_LapCor.

#### 2.2.1. Spectroscopic data for H_3_LapCor

Yield 0.040 g; 0.041 mmol (16%). M. p. > 300 °C (decomp.). ^1^H NMR (600 MHz, CDCl_3_): 1.48 (s, 6H, CH_3_, 1), 2.03 (s, 2H, CH_2_, 2), 3.17 (s, 2H, CH_2_, 3), 7.44 (s, 1H, Ar_Lap_, 4), 7.54 (s, 1H, Ar_Lap_, 7), 8.25 (d, 2H, *J* = 7.6 Hz, Ar_LapCor_, 8), 8.31 (d, 2H, *J* = 8.4 Hz, Ar_LapCor_, 9), 8.47 (d, 2H, *J* = 7.7 Hz, Ar_Lap_, 5, 6), 8.55 (d, 2H, *J* = 4.1 Hz, β-H_corrole_, 12, 14), 8.66 (s, 4H, β-H_corrole_, 10, 11) and 9.09 (d, 2H, *J* = 4.05 Hz, β-H_corrole_, 13, 15). ^19^F NMR (565 MHz, CDCl_3_): −137.86 (s, 2F, F_*meta*_); −152.77 (t, 1F, *J* = 21.5 Hz, F_*para*_) and −161.74 (d, 2F, *J* = 23.35 Hz, F_*ortho*_). FTIR (ATR, cm^−1^): 3423, 3093, 2920, 2851, 1715, 1486, 1097, 954, 785. HRMS-ESI(+): *m/z* = 957.2394 (calcd for C_53_H_30_F_10_N_6_O); *m/z* = 957.2316 ([M + H]^+^, found for C_53_H_30_F_10_N_6_O).

### 2.3. Absorption and emission analysis

UV-vis absorption spectra in all solvents (toluene – Tol; tetrahydrofuran – THF; dichloromethane – DCM; ethanol – EtOH; acetonitrile – ACN; *N*,*N*-dimethylformamide – DMF, dimethyl sulfoxide – DMSO and DMSO(5%)/Tris–HCl pH 7.4 buffer mixture) were obtained using the Shimadzu UV-2600 spectrophotometer (1.0 cm optical path length) and measured in the 250–800 nm region, with concentrations of about 10 μM.

For steady-state fluorescence emission spectra, were obtained using the Horiba Jobin-Yvon Fluoromax Plus fluorimeter (slit Em/Exc 2.0 mm), measured in the 600–800 nm region, with concentrations of about 5.0 μM, and excited in the respective Soret band. The fluorescence quantum yields (*Φ*_f_) of the corrole sample dissolved all solvents were measured using *meso*-tetra(phenyl)porphyrin in DMF solution as standard (TPP; *Φ*_f_ = 11%).^14^ Unfortunately, it was not possible to record any emission spectrum of corrole in DMSO (5%)/Tris–HCl pH 7.4 buffer mixture solution, due to the absence of emission bands.

Once the steady-state fluorescence spectra were recorded for all the samples, fluorescence quantum yields were determined using adequate equation according to the current literature.^15^ Lifetime fluorescence decays (*τ*_f_) in the singlet-excited state were recorded using Time Correlated Single Photon Counting (TCSPC) method with DeltaHub controller in conjunction with Horiba fluorimeter. Data was processed with DAS6 and Origin® 8.5 software using exponential (mono-exponential) fitting of raw data. NanoLED (Horiba) source (1.0 MHz, pulse width <1.3 ns at 441 nm excitation wavelength) was used as an excitation source. In this way, radiative (*k*_r_) and non-radiative (*k*_nr_) constants can be determined by knowing the fluorescence quantum yield and fluorescence lifetime, as following equations according to the literature.^16^

### 2.4. TD-DFT calculations

Electronic and structural properties of the corrole were studied through DFT calculations, as implemented in Gaussian 09.^17^ The ground state geometrical structures were optimized (energy minimization) with Berny's optimization algorithm, which involves calculating the analytical derivatives of the energy with respect to nuclear coordinates, specifically in redundant internal coordinates.^17^ The optical absorption spectra were obtained by a single-point TDDFT calculation on the optimized geometries. All calculations were conducted at the wB97XD/6-31G(d,p) level of theory.^18^ The polarizable continuum model (PCM) was employed to calculate molecular properties in dichloromethane (DCM), dimethyl sulfoxide (DMSO), or acetonitrile (ACN).

### 2.5. Electrochemical measurements

Cyclic voltammograms were recorded with a potenciostat/galvanostat AutoLab Eco Chemie PGSTAT 128 N system at room temperature and under argon atmosphere, in dry DCM solution. Electrochemical grade tetrabutylammonium hexafluorophosphate (TBAPF_6_, 0.1 M) was used as a supporting electrolyte. Employing a standard three-component system these CV experiments were carried out with: a glassy carbon working electrode; a platinum wire auxiliary electrode and a platinum wire pseudo-reference electrode. To monitor the reference electrode, the ferrocenium/ferrocene redox couple was used as an internal ref. 19.

### 2.6. Aggregation and stability in solution

UV-vis absorption spectroscopy was conducted as a function of successive increase of corrole concentration (0.5 to 20 μM) in ACN, DMF, DMSO and DMSO(5%)/Tris–HCl mixture buffer (pH 7.4) solutions and changes in the respective Soret band were monitored.^20^ The stability experiments in ACN, DMF, DMSO and DMSO(5%)/Tris–HCl mixture buffer (pH 7.4) of related corrole was also monitored by absorption electronic UV-vis measurements at total time of 3 days. All experiments were performed in duplicate and independently.

### 2.7. Photostability, singlet oxygen quantum yield (*Φ*_Δ_) and superoxide formation assays

The photostability experiments in DMSO(5%)/Tris–HCl mixture buffer (pH 7.4) solution of related corrole was also monitored by UV-vis absorption analysis at different exposure times (0 to 30 min) under white-light LED array system (400 to 800 nm) at irradiance of 50 mW cm^−2^ and a total light dosage of 90 J cm^−2^ and red-light LED array system (660 nm) at irradiance of 100 mW cm^−2^ and a total light dosage of 180 J cm^−2^, respectively. Photodegradation constant (*k*_PB_) and photodegradation quantum yield (*Φ*_PB_) are also determined. All experiments were performed in duplicate and independently.

Singlet oxygen production (*Φ*_Δ_) was recorded according to typical 1,3-diphenylisobenzofuran (DPBF) photo-oxidation analysis, in DMSO, DMF and ACN solutions. In order to measure singlet oxygen generation, absorption UV-vis spectra of each solution were recorded at different exposure times (0 to 900 s, using red-light LED array system (660 nm) at irradiance of 100 mW cm^−2^ and a total light dosage of 180 J cm^−2^). The singlet oxygen production quantum yield (*Φ*_Δ_) was calculated applying according to the literature, using as standard *meso*-tri(phenyl)corrole TPhCor in DMSO, *Φ*_Δstd_ = 0.67.^21^

The superoxide formation in DMF and DMSO solutions was evaluated by NBT reduction experiment, to detect the formation of superoxide radical species (O_2_˙^−^). This approach was carried out using at the same conditions in the literature.^22^ Corrole sample was irradiated under aerobic conditions with white-light LED source at total period of 30 min (irradiance of 50 mW cm^−2^ and a total light dosage of 90 J cm^−2^). The progress of the reaction was monitored by following the increase of the absorbance close to 560 nm. The superoxide generation constant (*k*_SO_) can be obtained from the slope in the plot Abs_formazan_*versus* time.

### 2.8. Water/*n*-octanol partition coefficients (log *P*_OW_)

The partition coefficient of corroles was determined using *n*-octanol (3.0 mL) and water (3.0 mL), according to the literature^23^ and the log *P*_OW_ value was calculated from UV-vis absorption analysis.

## 3 Results and discussions

### 3.1. Corrole synthesis

**Scheme 2:**
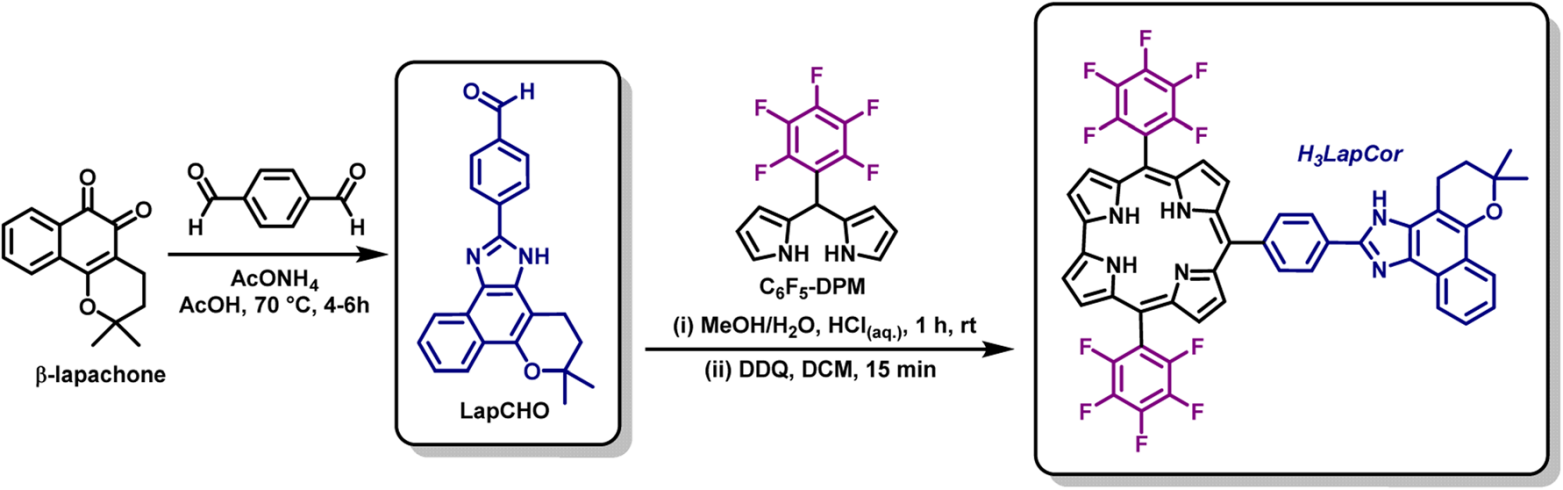
Synthetic route for the synthesis of corrole H_3_LapCor.

The access to *meso*-10-substituted corrole H_3_LapCor was performed, according to Gryko methodology,^24^ and involved the condensation of (pentafluorophenyl) dipyrromethane C_6_F_5_-DPM and the derivative prepared from β-lapachone LapCHO,^5^ in a mixture of MeOH/H_2_O using HCl as catalyst (Scheme 2). The reaction mixture was maintained under stirring for 1 h at room temperature and after the usual work up and chromatographic purification, we were able to isolate corrole H_3_LapCor in 16%. Furthermore, condensation in DCM in the presence of TFA acid was tested, but the yields were not satisfactory (close to 3.0% – data not show). The structure of this free-base corrole was fully confirmed by mass spectrometry, NMR, electrochemistry, and absorption/emission spectroscopy.

### 3.2. Structural characterization of corrole

Considering the characterization of compound H_3_LapCor, its mass spectrum (see ESI – Fig. S1†) show three ion peaks: one at *m/z* 957, corresponding to the original ion ([M + H]^+^), other at *m/z* 901 probably corresponding to the species originating from the fragmentation of the aliphatic ring of lapachol rings and other at *m/z* 685 probably corresponding to loss of lapachol unit followed by HF loss of corrole substituent. In the FT-IR ATR vibrational spectrum, the main vibrations of the corrole were selected, due to the difficult attribution of the signals with precision (see ESI – Fig. S2†). In general, the ^1^H NMR spectrum of corrole H_3_LapCor showed the expected profile of typical *trans*-A_2_B-corroles (see ESI – Fig. S3†). For example, the signals at *δ* 9.10 to 8.50 ppm range correspond to the β-pyrrolic protons of corrole derivative. As expected, the doublets at *δ* 8.35 to 8.20 ppm range were identified as the phenyl ring protons between corrole and lapachol moiety. The resonances of the protons of the phenyl rings of lapachol were assigned to the signals at *δ* 8.47, 7.54, and 7.44 ppm, respectively. Finally, the aliphatic proton signals corresponding to the O-ring are observed at *δ* 3.20 to 1.40 ppm range. The ^1^H–^1^H COSY 2D and ^1^H–^13^C HMBC NMR spectra are listed in the ESI (Fig. S4 and S5†).

The ^19^F NMR spectrum of H_3_LapCor showed the same profile of the typical C_6_F_5_-substituted corroles.^25^ For example, ^19^F NMR spectrum shows the resonances of the fluorine atoms in the *meta* position as a singlet signal at *δ* −137.86 ppm, the resonances of *para* fluor atoms appear as triplet at *δ* −152.77 ppm and the ones belonging to *ortho* atoms as doublet at *δ* −161.74 ppm (see ESI – Fig. S6†).

### 3.3. Photophysical properties of corroles

**Fig. 1:**
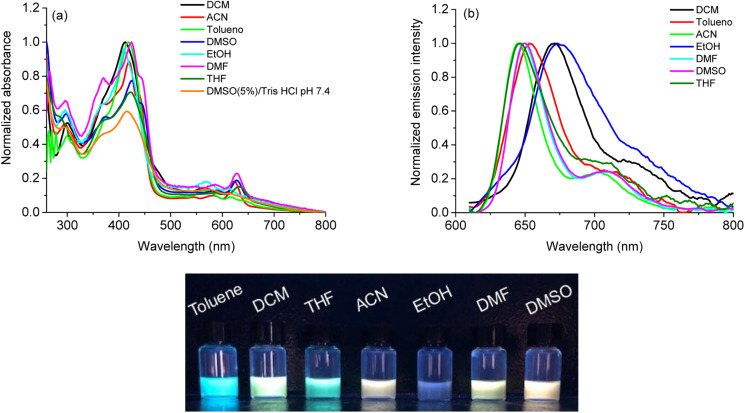
(a) Normalized absorption UV-vis spectra of corrole H3LapCor in all solvents (concentration at 10 μM), (b) steady-state fluorescence emission spectra of corrole H_3_LapCor in all solvents (concentration at 5.0 μM), using *λ*_exc_ the Soret band and (c) solutions in UV_365nm_ light irradiation.

The absorption UV-vis spectra of corrole H_3_LapCor was recorded in several different solvents: acetonitrile (ACN), dichloromethane (DCM), dimethyl sulfoxide (DMSO), DMSO(5%)/Tris–HCl pH 7.4 buffer mixture, *N*,*N*-dimethylformamide (DMF), ethanol (EtOH), tetrahydrofuran (THF) and toluene (Tol) at room temperature and 250–800 nm range and absorption spectra illustrated in Fig. 1A.

For compound H_3_LapCor, maximum absorption bands were observed at about 419 to 425 nm, which are attributed to the Soret band in the corrole structure. The changes in the absorption maxima in this transition have a small variation, depending on the polarity of the solvent, and the values are listed in Table 1. The Q-bands were found between 560 to 630 nm range, with corrole showing more intense Q bands in the red region in some solvents, such as ACN, DMF and DMSO (Fig. 1A). According to the current literature, the corrole derivatives here can be present in the anionic form in some solution and this possibility can affect the UV-vis profile in lower energy region, due to the basicity of the solvent.^26^ Also, at more higher energy region, the transition bands around 280 to 300 nm which can be assigned to the lapachol moiety (π → π*).

**Table 1:** Photophysical parameters of the studied corrole in all solvents

Solvent^a^	λ, nm (ε; M^−1^ cm^−1^)^b^	*λ*_em_, nm (*Φ*_f_, %)^c^^,^^d^	SS e(nm cm^−1−1^)	*τ*_f_ f(ns)	*χ* ^2^	*k*_r_ (×10^6^ s^−1^)^g^	*τ*_r_ h(ns)	*k*_nr_ (×10^6^ s^−1^)^g^
Toluene	300 (26 732), 419 (59 182), 566 (8151), 617 (5939)	653 (3.0)	36/893	4.71 ± 0.05	1.02591	6.37	1.57	205.9
THF	367 (39 098), 421 (52 165), 588 (7237), 631 (10 100)	646 (2.3)	15/368	4.32 ± 0.04	1.14370	5.32	1.88	226.1
DCM	299 (37 313), 412 (70 099), 566 (10 820), 611 (8681)	671 (2.0)	60/1463	4.19 ± 0.04	1.07256	4.77	2.10	233.8
EtOH	296 (40 248), 409 (65 373), 567 (10 945), 612 (7985)	674 (4.1)	62/1503	4.05 ± 0.05	1.06452	10.12	0.99	236.8
ACN	295 (41 829), 419 (71 884), 582 (7998), 623 (14 465)	645 (3.7)	22/547	4.40 ± 0.05	1.12275	8.41	1.19	218.8
DMF	297 (58 504), 425 (89 998), 586 (14 659), 628 (20 582)	649 (4.4)	21/515	4.20 ± 0.04	1.17435	10.48	0.95	227.6
DMSO	298 (55 812), 425 (74 389), 587 (12 335), 628 (18 235)	650 (7.0)	22/539	4.51 ± 0.05	1.02708	15.52	0.64	206.2

a Dieletric constant (*ε*_0_ = 2.38 for toluene; *ε*_0_ = 7.52 for THF; *ε*_0_ = 8.93 for DCM; *ε*_0_ = 24.6 for EtOH; *ε*_0_ = 36.64 for ACN; *ε*_0_ = 38.25 for DMF and *ε*_0_ = 47.0 for DMSO).

b Concentration at 10 μM.

c Concentration at 5.0 μM and excited in the first Q band.

d Using TPP in DMF solution as standard (*Φ*_f_ = 11%).

e SS = Stokes shifts (Q bands): Δ*λ* = 1/*λ*_abs_ − 1/*λ*_em_ (cm^−1^).

f Excited by nanoLED source at 441 nm.

g Radiative and non-radiative rate constants.

h Radiative lifetimes.

The steady-state fluorescence emission spectra of corrole H_3_LapCor was recorded in all solvents mentioned above, and the emission spectra and UV lamp solution photography are illustrated in Fig. 1B and C. The studied corrole exhibited classical emission bands at the red region when excited in Soret transition band, which can be assigned to the S_0_ → S_2_ electronic transition state (Table 1). Some emission shifts are observed in the maximum emission peaks, mainly in DCM and EtOH solution and we can attribute this to a difference in the stabilization of the structures in the excited state, probably by secondary interactive forces. The fluorescence quantum yields (*Φ*_f_) and Stokes Shifts (SS) are measured in all solvents (Table 1) and the results are low and agree with similar fluorescence properties of corrole derivatives in the literature.^27^ The small SS values were observed for all solvents, and this can be attributed to the vibrational relaxation or dissipation and solvent reorganization, which can increase the separation of the energy levels of the ground and excited states.

Using time-resolved fluorescence decay (*τ*_f_), the corrole H_3_LapCor presented slightly variation in the fluorescence decays according to the solvent polarity (Table 1). The fluorescence lifetimes directly excited in the Soret transitions are about 4.00 to 4.50 ns range, and this likely indicates a reduction in the non-radiative (*k*_nr_) relaxation due to more restricted molecular motion, thereby favoring radiative (*k*_r_) constant values (Table 1). The fluorescence lifetime measured for corrole are in agreement with those obtained for other corrole derivatives in the literature.^27^ Using the radiative decay time (*τ*_r_), it is possible to see that the is very slow when compared with the fluorescence lifetime in all solvents, indicating that probably for corrole H_3_LapCor the main relaxation mechanisms are probably the internal conversion (IC) and/or triplet state formation.

### 3.4. TD-DFT analysis

Fig. S7† (ESI) shows the TD-DFT optical absorption for the corrole structures in DCM (black), DMSO (red), and ACN (green). The data were obtained at the ground state equilibrium geometry for each compound.^28^ We can notice a very similar absorbance behavior between all the solvents, with a slight red shift observed in acetonitrile. As peculiar in corrole-conjugated molecules,^29^ we found two peaks at the Q-band (∼510 and ∼535 nm), which correspond to S_0_ → S_1_ and S_0_ → S_2_ transitions, and a more pronounced peak at approximately 370 nm in the Soret band, which correspond to an S_0_ → S_3_ transition. All the implicit environments present very close values for energy and oscillator strength in these transitions (see ESI; Table S1†).

**Fig. 2:**
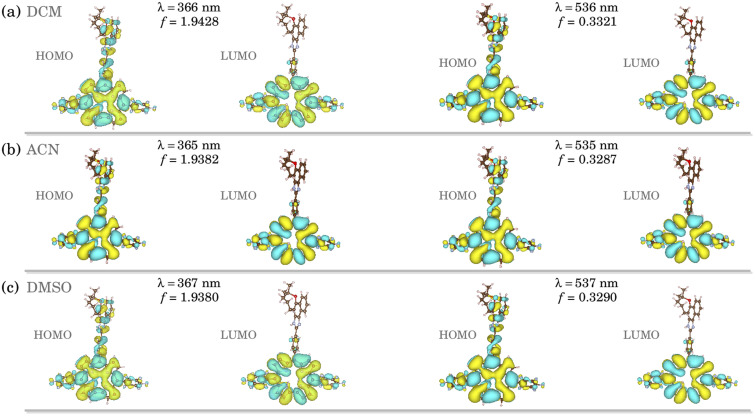
Natural transition orbitals (NTOs) for studied H_3_LapCor corrole associated with the absorption peaks in (a) DCM, (b) ACN and (c) DMSO.

To elucidate the optical transitions predicted by TD-DFT calculations, in Fig. 2 we show the natural transition orbitals (NTOs) associated with the most prominent peaks in the corrole compound, in the S_0_ → S_1_ and S_0_ → S_3_ transitions. As we can learn from Fig. 2, all the transitions associated with both the Soret and the Q-bands come from the entire molecule (HOMO) to the tetrapyrrole macrocycle portion of the corrole (LUMO). In both environments, the HOMO and LUMO orbitals are formed from π-like orbitals on the entire molecule, and do not exhibit a solvent-dependent electronic distribution. The solvent does not affect the optically induced electronic transitions in these corroles with lapachol moiety. The results highlight the electronic distribution in these compounds.

We found a HOMO–LUMO energy gap of 5.442, 5.355, and 5.354 eV for DCM, DMSO, and ACN implicit solvents, respectively. A higher HOMO–LUMO energy gap implies higher kinetic energy, with elevated chemical reactivity. The HOMO–LUMO energy gaps of all solvents are very similar, which implies that the stability is independent of the environment.

### 3.5. Electrochemical analysis

The electrochemical behavior of corrole H_3_LapCor was investigated by cyclic voltammetry (CV) analysis in dry DCM solution and the redox peaks are listed in the Table 2. All CV data are presented in the ESI (Fig. S8†).

**Table 2:** Redox potentials of corrole H_3_LapCor, in dry DCM solution (*E versus* SHE) at scan rate 100 mV s^−1^

Compound	*E*_ox1_	*E*_ox2_	*E*_ox3_	*E*_red1_	*E*_red2_	*E*_HOMO_ c(eV)	*E*_LUMO_ d(eV)	Δ*E*^e^
H_3_LapCor	+0.91 V^a^	+1.38 V^a^	+1.58 V^a^	−1.15 V^b^	−1.83 V^b^	−5.71	−3.65	2.06

a Anodic peak = *E*_pa_.

b Cathodic peak = *E*_pc_.

c *E*_HOMO_ = −[4.8 + *E*_ox1_ (*versus* SHE)] eV.

d *E*_LUMO_ = −[4.8 + *E*_red1_ (*versus* SHE)] eV.

e Δ*E* = *E*_HOMO_ – *E*_LUMO_.

Scanning in the anodic region (positive potentials) the free-base corrole H_3_LapCor showed three irreversible processes at +1.00 to +1.60 V range. These processes can be assigned to the π-cation radical species, following to the cationic and dicationic species, according related to the literature.^30^ Moving to the cathodic region (negative potentials) studied corrole presented two irreversible processes at −1.10 to −1.85 V range. The observed redox processes can be assigned to the π-anion radical species, following to the anionic and dianionic species.

### 3.6. Photobiological experiments

#### 3.6.1. Aggregation, stability in solution, photostability and partition coefficients

**Fig. 3:**
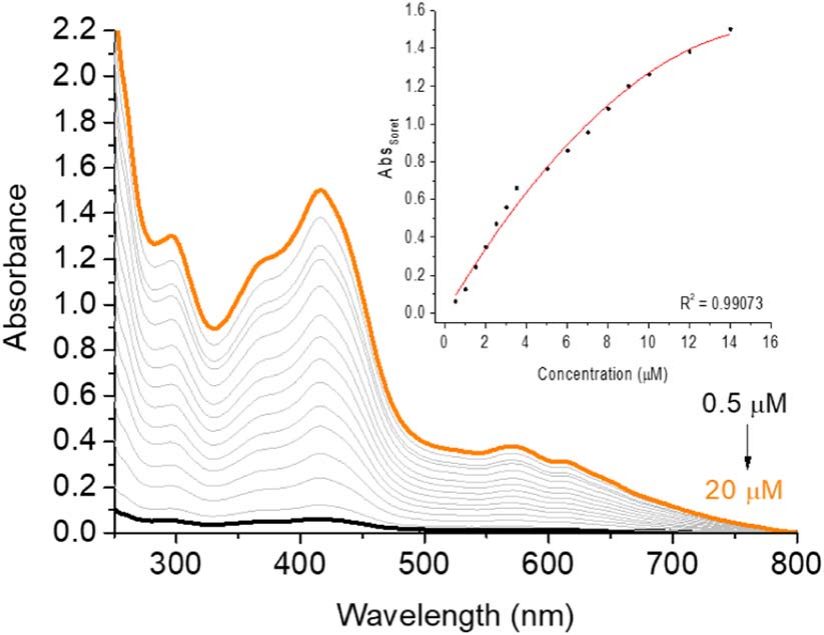
UV-vis aggregation assays of corrole H_3_LapCor in DMSO(5%)/Tris–HCl pH 7.4 buffer mixture solution, in the concentration variation from 0.5 to 20 μM. Inset: Abs_Soret_*versus* concentration plot.

To evaluate the aggregation behavior of corrole H_3_LapCor in solution, we studied this phenomenon by UV-vis absorption analysis in several solvents (ACN, DMF, DMSO and DMSO(5%)/Tris–HCl pH 7.4 buffer mixture). In all cases, we observed that there was not a significant shift at the maximum Soret absorbance wavelengths. Using the DMSO (5%)/Tris–HCl pH 7.4 buffer mixture solution as example, an increase was observed in the absorption peaks as a function of the concentration variation from 0.5 to 20 μM (Fig. 3). In addition, no new absorption band on the UV-vis region was observed in any cases, indicate that aggregation is not present. All absorption spectra in other solvents are listed in the ESI (see Fig. S9–S11†).

**Fig. 4:**
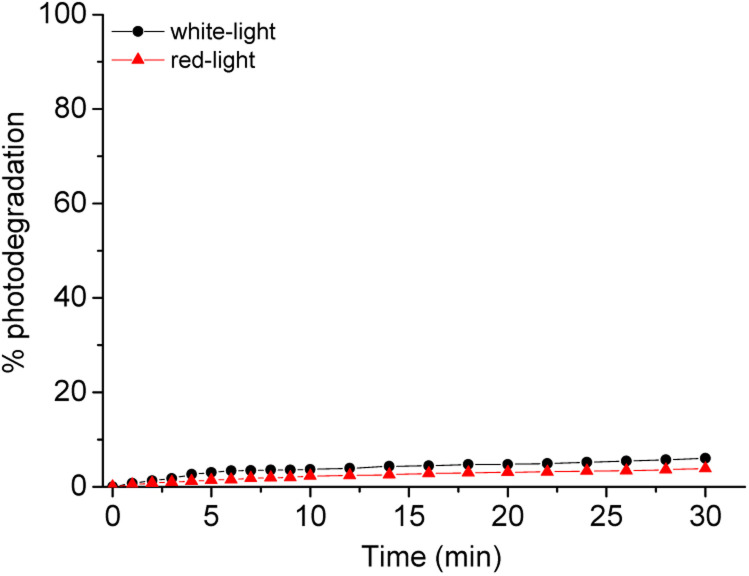
Comparative photostability behavior of corrole H_3_LapCor in white-light (black line) and red-light (red line) irradiation conditions, after 30 min.

The difference in relation to the photostability of the corrole is described in Fig. 4. The absorption spectra of corrole under white and red-light LED irradiation conditions (white-LED with irradiance of 50 mW cm^−2^ and a total light dosage of 90 J.cm^−2^; red-LED with irradiance of 100 mW cm^−2^ and a total light dosage of 180 J.cm^−2^, both at period of 30 min) in DMSO(5%)/Tris–HCl buffer pH 7.4 buffer mixture solution show a similar photo-bleaching profile (Fig. 4 and Table 3). This may be attributed to better stabilization of the pentafluorophenyl (C_6_F_5_) groups in the *meso* macrocycle positions. In this way, the presence of C_6_F_5_ groups increases the photostability of the corrole, which was also observed in other corrole derivatives in the literature.^27^ Moreover, no changes in the profile of the electronic spectra of corrole was observed, possibly indicating that there was no formation of by-products under photo-bleaching conditions (see ESI – Fig. S12 and S13†).

Photobiological parameters of corrole H_3_LapCor

**Table:** 

Solvent	^1^O_2_ generation		O_2_˙^−^ generation
	*k*_po_ a(M^−1^ s^−1^)	*Φ*_Δ_ (%)	*k*_SO_ b(M^−1^ s^−1^)
ACN	2.46 × 10^−4^	21.0	—
DMF	4.96 × 10^−4^	45.0	3.50 × 10^−3^
DMSO	3.39 × 10^−4^	35.0	3.98 × 10^−3^

a DPBF photooxidation constant, using *meso*-tri(phenyl)corrole TPhCor as standard in DMSO solution (*k*_po_ = 6.50 × 10^−4^ M^−1^ s^−1^ and *Φ*_Δ_ = 67%).^30^

b Superoxide formation constant, by NBT reduction assay in DMSO and DMF solution.

c Photobleaching constant determined by photostability assays (white-LED with irradiance of 50 mW cm^−2^ and a total light dosage of 90 J cm^−2^ for 30 min; red-LED with irradiance of 100 mW cm^−2^ and a total light dosage of 180 J cm^−2^ for 30 min), in DMSO(5%)/Tris–HCl pH 7.4 buffered mixture solution.

d Photobleaching quantum yield determined by photostability assays (white-LED with irradiance of 50 mW cm^−2^ and a total light dosage of 90 J cm^−2^ for 30 min; red-LED with fluence rate of 100 mW cm^−2^ and a total light dosage of 180 J cm^−2^ for 30 min), in DMSO(5%)/Tris–HCl pH 7.4 buffered mixture solution.

**Table:** 

	Photobiological parameters
Log *P*_OW_	+2.31
*k*_PB_ (min^−1^)^c^ (white-light)	1.96 × 10^−3^
*Φ*_PB_ (%)^d^ (white-light)	4.00
*k*_PB_ (min^−1^)^c^ (red-light)	1.46 × 10^−3^
*Φ*_PB_ (%)^d^ (red-light)	2.80

Furthermore, the partition coefficients (log *P*_OW_) were measured for corrole derivative in octanol/water mixture and the value found for the H_3_LapCor is in accordance with the literature, with neutral corrole derivatives,^31^ showing a hydrophobic character, by positive log *P*_OW_ values (Table 3).

#### 3.6.2. ROS generation assays

The ability of corrole H_3_LapCor to produce reactive oxygen species (ROS), in this case, singlet oxygen (^1^O_2_), was monitored using 1,3-diphenylisobenzofuran (DPBF) quencher in ACN, DMF and DMSO solution.^30,31^ The DPBF method has been widely used to provide a quantitative analysis of singlet oxygen production by ^1^O_2_ quantum yield (*Φ*_Δ_). The DPBF photo-oxidation UV-vis spectra in ACN and DMSO are presented in the ESI (Fig. S14 and S15†).

**Fig. 5:**
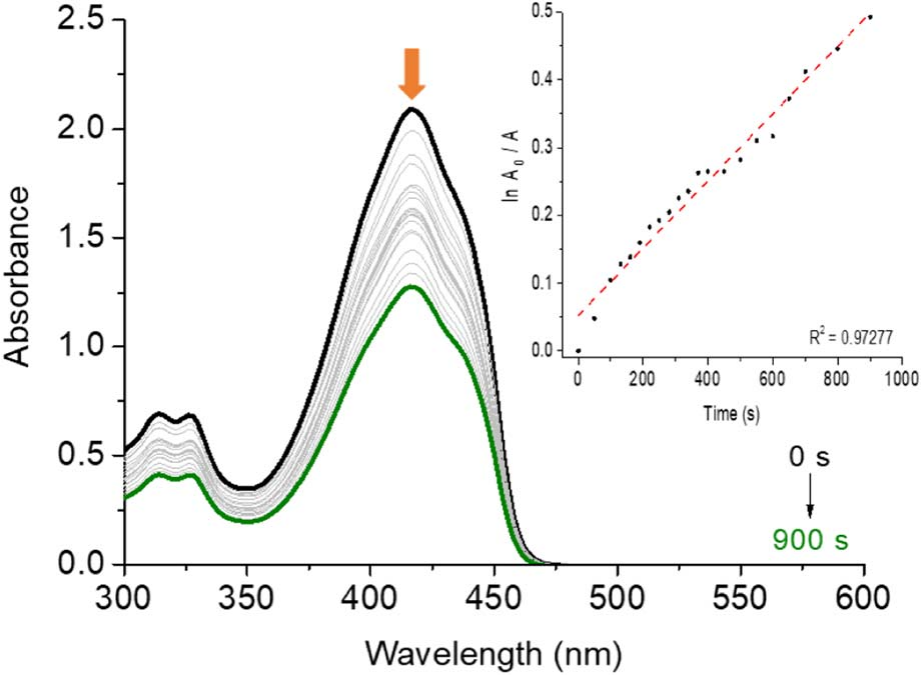
DPBF photo-oxidation experiment in DMF solution by irradiation with red-light LED source (660 nm; irradiance of 100 mW cm^−2^ and a total light dosage of 90 J cm^−2^) in the presence of corrole H_3_LapCor. The inset shows the first order kinetic profile.

In the Table 3 are presented the *Φ*_Δ_ and photo-oxidation constants (*k*_po_) values in three different solvents of corrole H_3_LapCor. As example, Fig. 5 shows the decrease in the DPBF absorbance, monitored at 415 nm during irradiation with a red-light LED source (660 nm), for corrole H_3_LapCor in DMF solution. The observed results show that corrole can produce singlet oxygen with higher yields in DMF and DMSO solution. This fact may be linked to the fact that this derivative in the related solutions has a greater tendency to population of triplet-excited states, favored by a possible intersystem crossing.^30–32^

The capacity of corrole H_3_LapCor to generate superoxide species (O_2_˙^−^) by type I pathway was investigated in DMF and DMSO solutions, and the superoxide formation constant (*k*_SO_) are presented in the Table 3. For this application, solutions of corrole containing NBT and the reducing agent NADH were irradiated with white-light LED source under aerobic conditions. The reaction can be monitored following the absorption band of this product, which is centered close to 560 nm (Fig. S16 and S17†). These results indicate studied corrole H_3_LapCor, after white-light irradiation conditions, can form O_2_˙^−^ in the presence of an electron donor agent (NADH), in both solutions. This result agrees with the data found for other corrole derivatives.^32^

## 4 Conclusions

A new *trans*-A_2_B-corrole derivative was synthesized from the reaction between a highly fluorescent lapachone derivative LapCHO and 2,3,4,5,6-(pentafluorophenyl)dipyrromethane C_6_F_5_-DPM. TDDFT calculations showed that the optical transitions are from the whole structure to the main corrole ring, indicating a contribution of the lapachone unit in all the solvent studied here. Furthermore, the predicted optical absorption is in good agreement with the experiment. Analysis of the photophysical and photobiological features of the newly compound revealed that this derivative presents some indispensable parameters from a good sensitizer in photodynamic processes like photostability, hydrophobic character and ROS generation.

## Author contributions

B. M. R., E. N. S. J. and B. A. I. idealized the work. B. M. R., C. C. D. G. P. B. and L. A. M. conducted the characterization, photophysical and photobiological experiments. V. N. R. and M. H. K. conducted the TD-DFT calculations. B. A. I., E. N. S. J. and M. H. K. wrote and correct the manuscript.

## Conflicts of interest

The authors declare that they do not have conflict of interest.

## Supplementary Material

RA-013-D3RA00823A-s001
