# Supplementary material for: First report of trans-A2B-corrole derived from a lapachone derivative: photophysical, TD-DFT and photobiological assays
Source: RSC Adv. 2023 Apr 11;13(16):11121–9. doi: 10.1039/d3ra00823a (PMC10089255; doi:10.1039/d3ra00823a)
Supplement: RA-013-D3RA00823A-s001 [file RA-013-D3RA00823A-s001.pdf]

Electronic Supplementary Material (ESI) for RSC Advances.
This journal is © The Royal Society of Chemistry 2023

Electronic Supplementary Information

First report of trans-A2B-corrole derived from a lapachone derivative: Photophysical, TD-DFT and Photobiological assays

Bruna M. Rodrigues,[a] Carlos C. Diniz,[a] Vinicius N. da Rocha,[b] Mateus H. Kohler,[b] Guilherme P. Brandão,[c] Luana A. Machado,[c,d] Eufranio N. da Silva Júnior*[c] and Bernardo A. Iglesias*[a]

[a]*Bioinorganic and Porphyrinoid Material Laboratory, Department of Chemistry, Federal University of Santa Maria, Santa Maria-RS, Brazil;*

[b]*Department of Physics, Federal University of Santa Maria, Santa Maria-RS, Brazil;*

[c]*Department of Chemistry, Federal University of Minas Gerais, Belo Horizonte-MG, Brazil;*

[d]*Julius Maximilians-Universität Würzburg (JMU), Institute for Inorganic Chemistry, Am Hubland, 97074 Würzburg, Germany.*

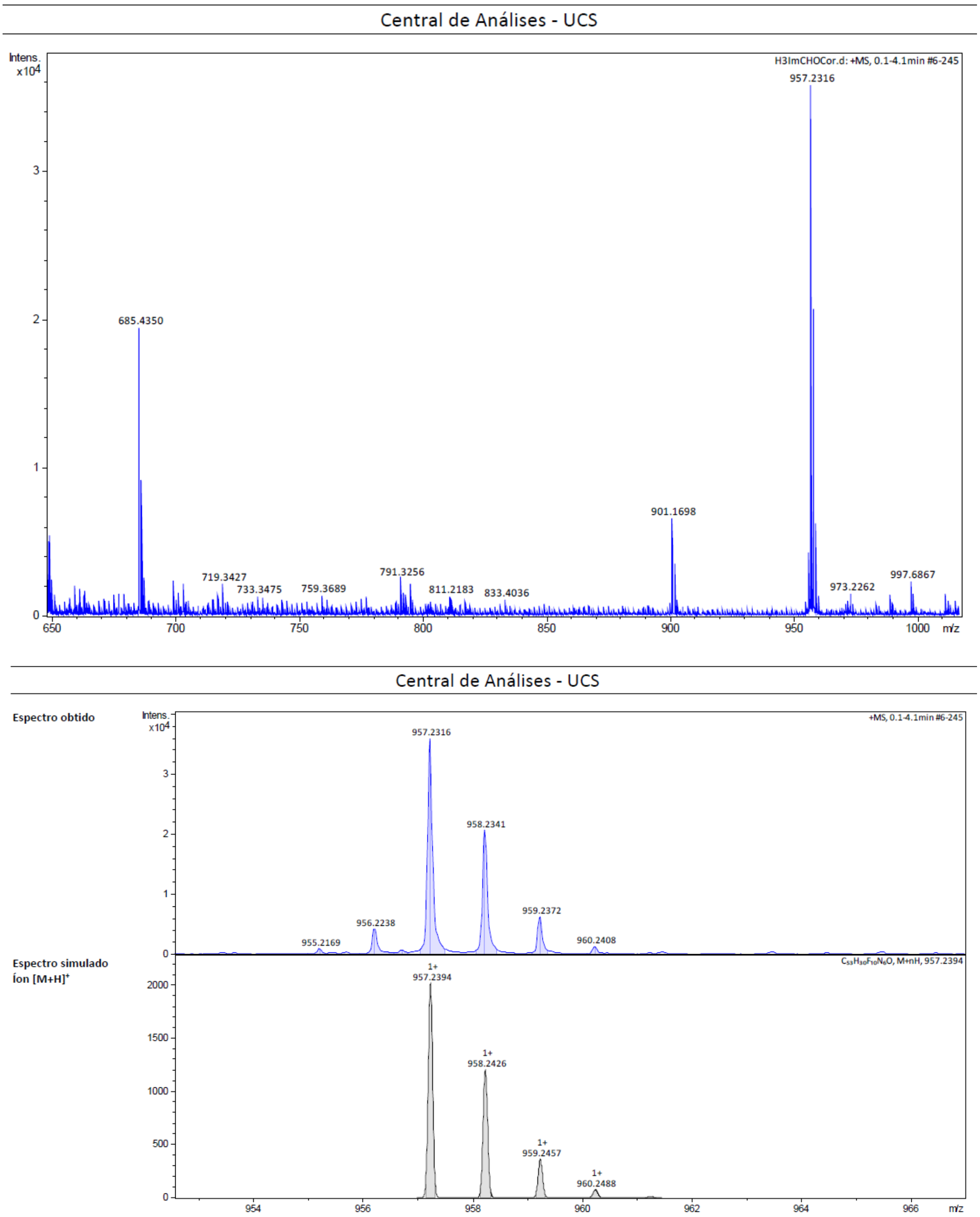

Figure S1. HRMS-ESI(+) spectrum of corrole **H_3LapCor**.

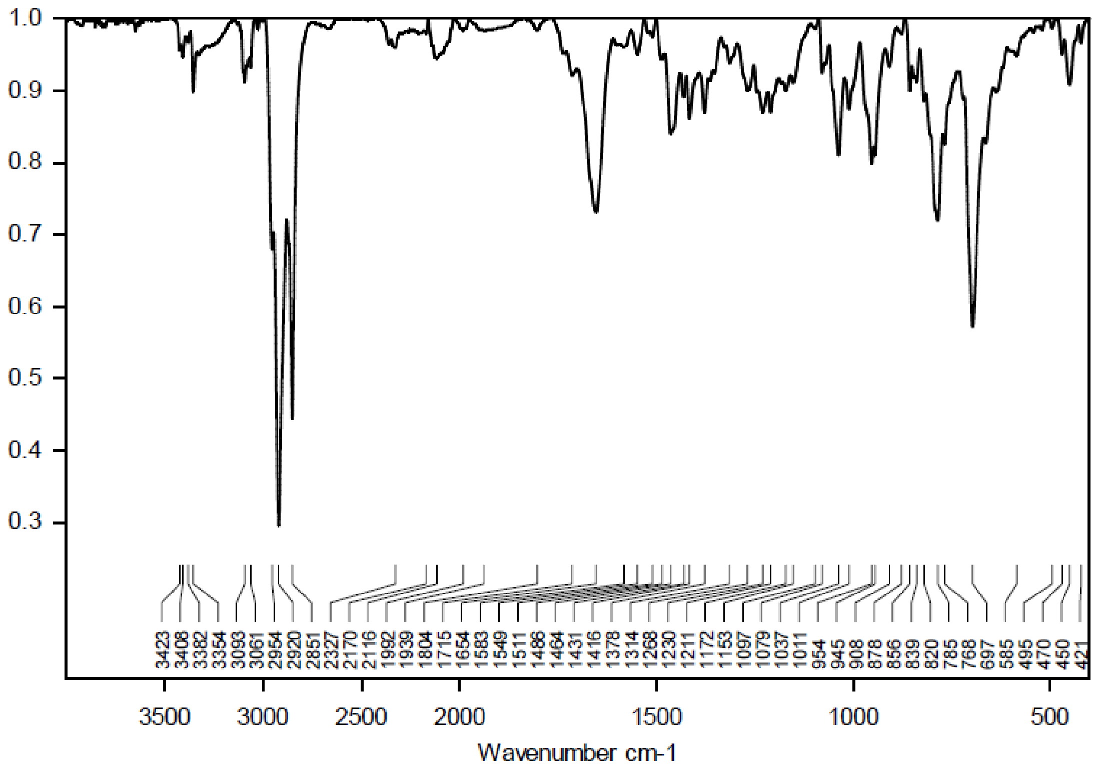

Figure S2. FTIR ATR spectrum of corrole $\mathbf{H_3LapCor}$.

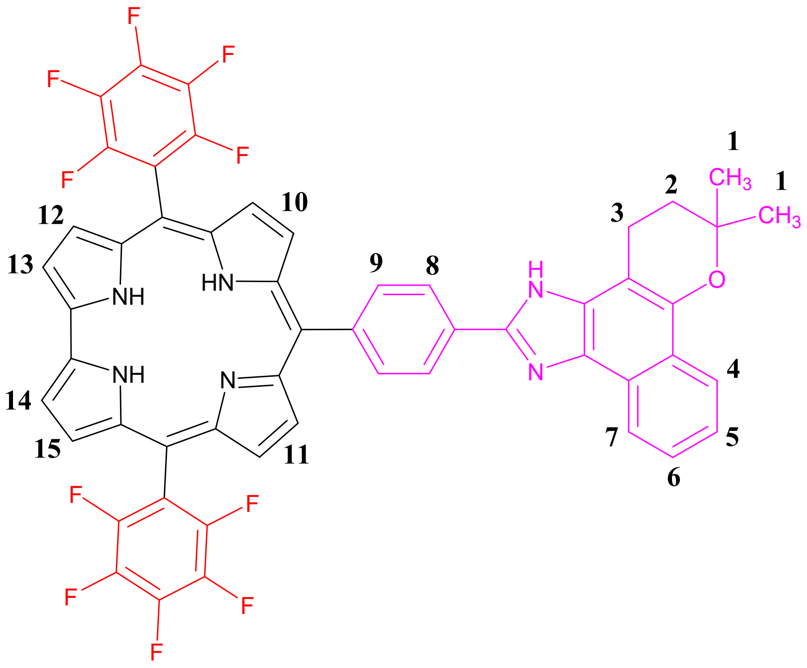

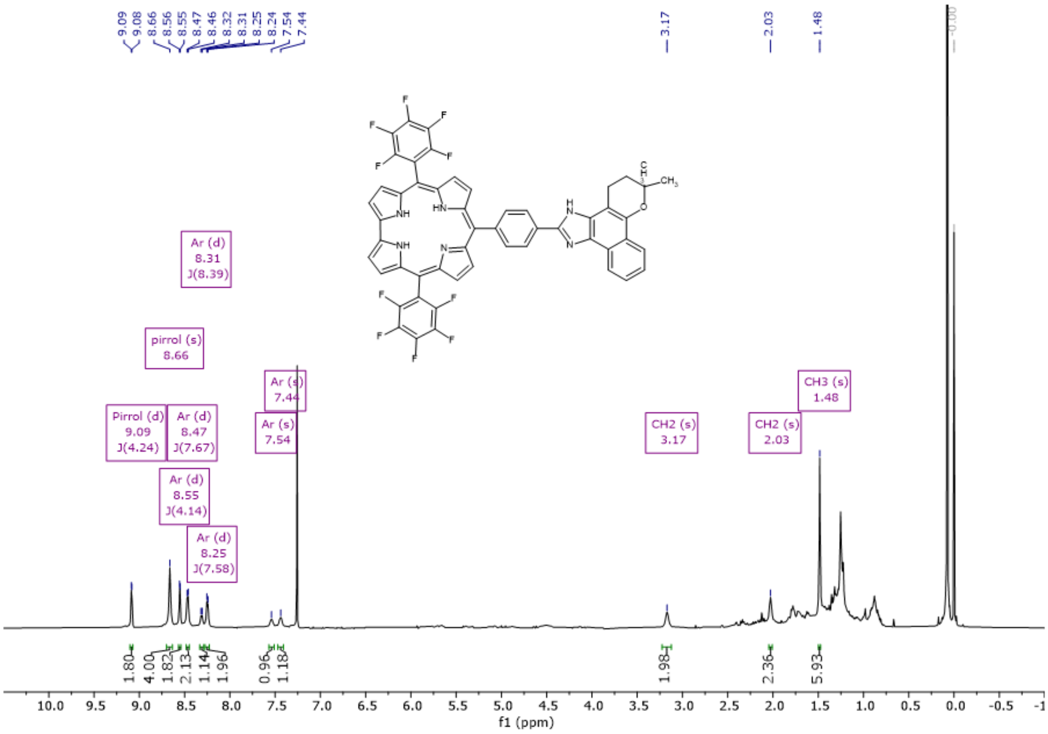
9.09
9.08
8.66
8.56
8.55
8.47
8.46
8.32
8.31
8.25
8.24
7.54
7.44
3.17
2.03
1.48
0.00
Ar (d) 8.31 J(8.39)
pirrol (s) 8.66
Ar (s) 7.44
Ar (s) 7.54
Pirrol (d) 9.09 J(4.24)
Ar (d) 8.47 J(7.67)
Ar (d) 8.55 J(4.14)
Ar (d) 8.25 J(7.58)
CH2 (s) 3.17
CH2 (s) 2.03
CH3 (s) 1.48
1.80
4.00
1.82
2.13
1.14
1.96
0.96
1.18
1.98
2.36
5.93
f1 (ppm)

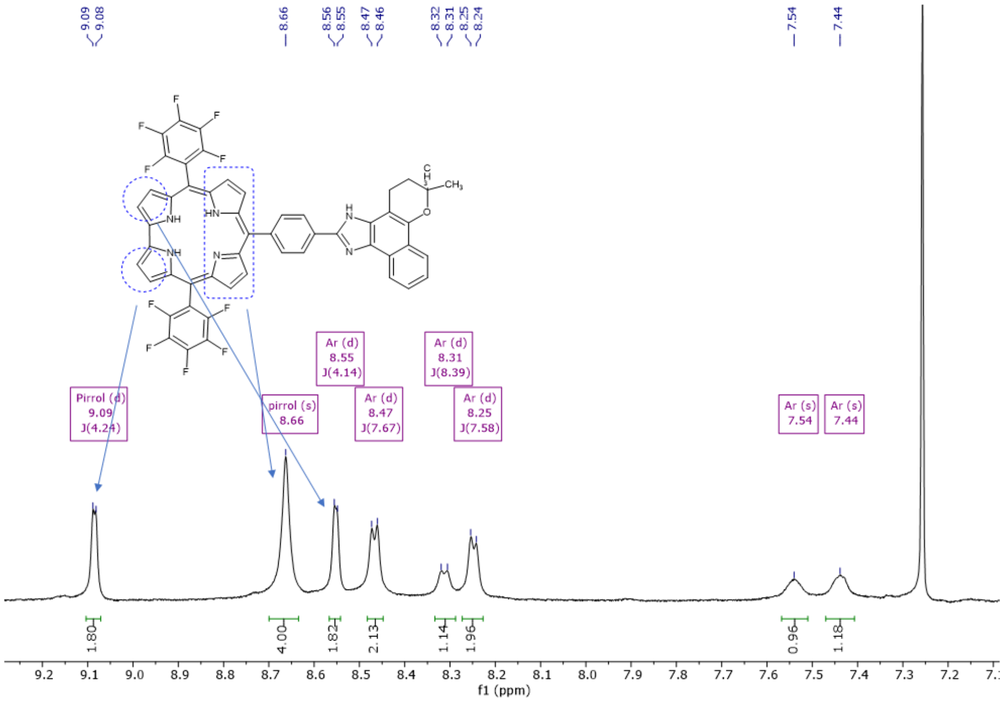
9.09
9.08
8.66
8.56
8.55
8.47
8.46
8.32
8.31
8.25
8.24
7.54
7.44
Pirrol (d) 9.09 J(4.24)
pirrol (s) 8.66
Ar (d) 8.55 J(4.14)
Ar (d) 8.47 J(7.67)
Ar (d) 8.31 J(8.39)
Ar (d) 8.25 J(7.58)
Ar (s) 7.54
Ar (s) 7.44
1.80
4.00
1.82
2.13
1.14
1.96
0.96
1.18
f1 (ppm)

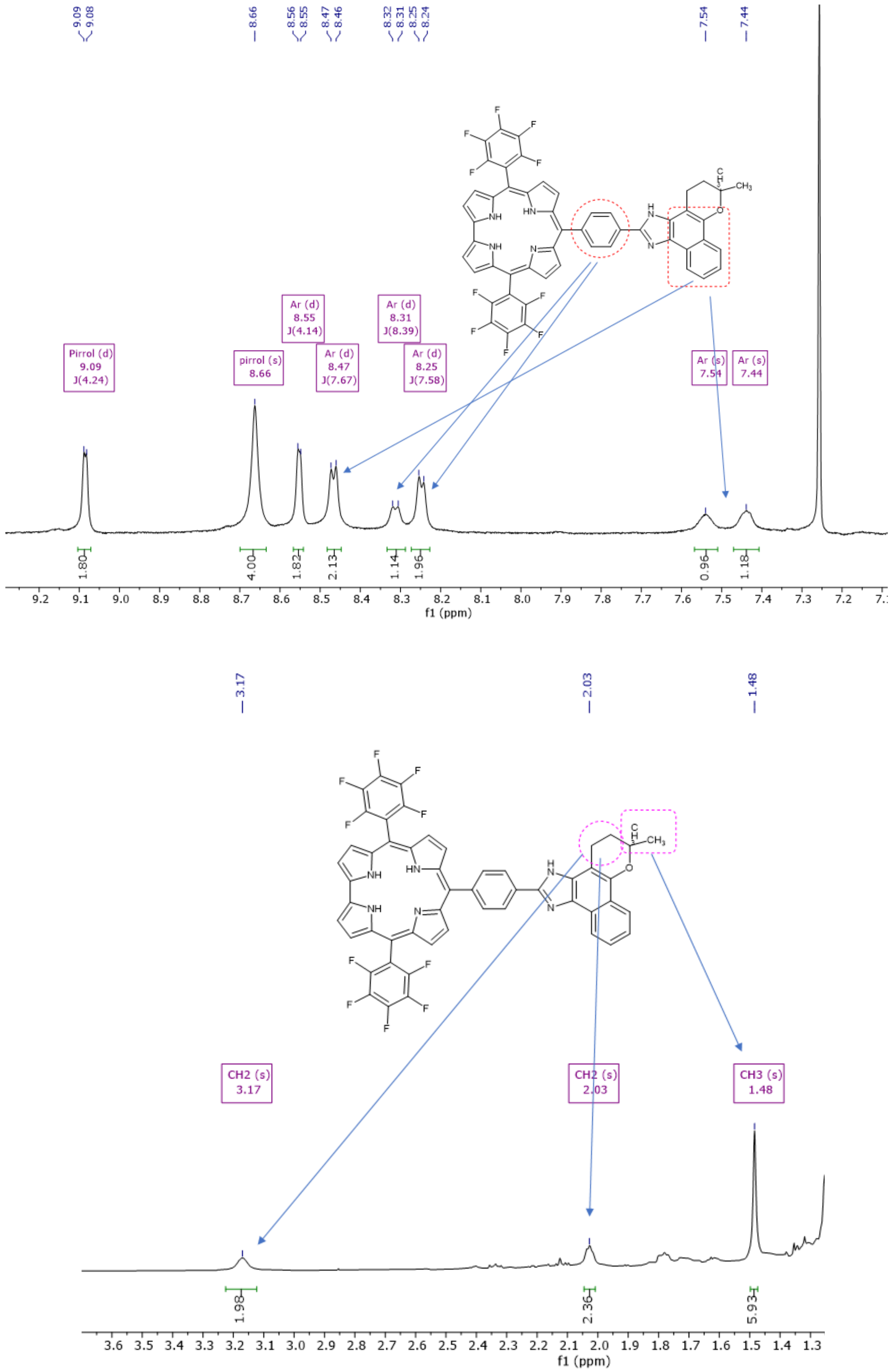

Figure S3. ^{1}H NMR spectrum (600 MHz) of corrole **H_3LapCor** in $CDCl_3$.

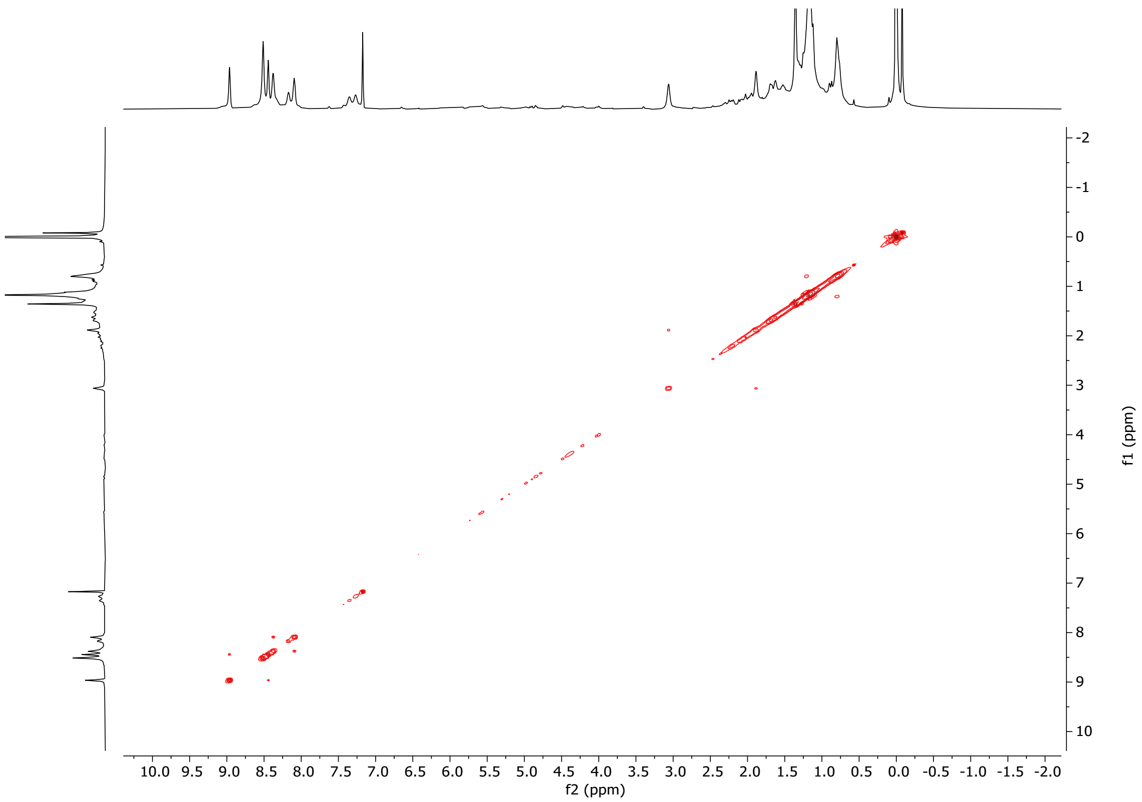

f2 (ppm)
f1 (ppm)
10.0 9.5 9.0 8.5 8.0 7.5 7.0 6.5 6.0 5.5 5.0 4.5 4.0 3.5 3.0 2.5 2.0 1.5 1.0 0.5 0.0 -0.5 -1.0 -1.5 -2.0
-2 -1 0 1 2 3 4 5 6 7 8 9 10

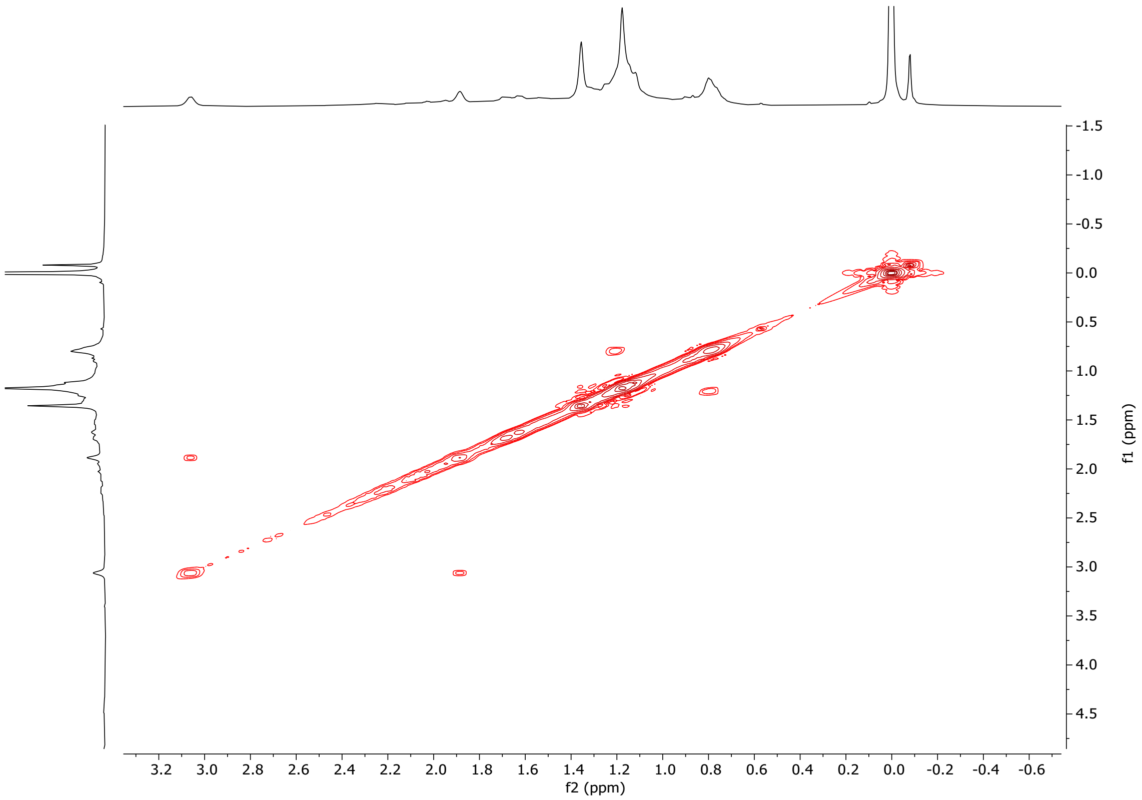

f2 (ppm)
f1 (ppm)
3.2 3.0 2.8 2.6 2.4 2.2 2.0 1.8 1.6 1.4 1.2 1.0 0.8 0.6 0.4 0.2 0.0 -0.2 -0.4 -0.6
-1.5 -1.0 -0.5 0.0 0.5 1.0 1.5 2.0 2.5 3.0 3.5 4.0 4.5

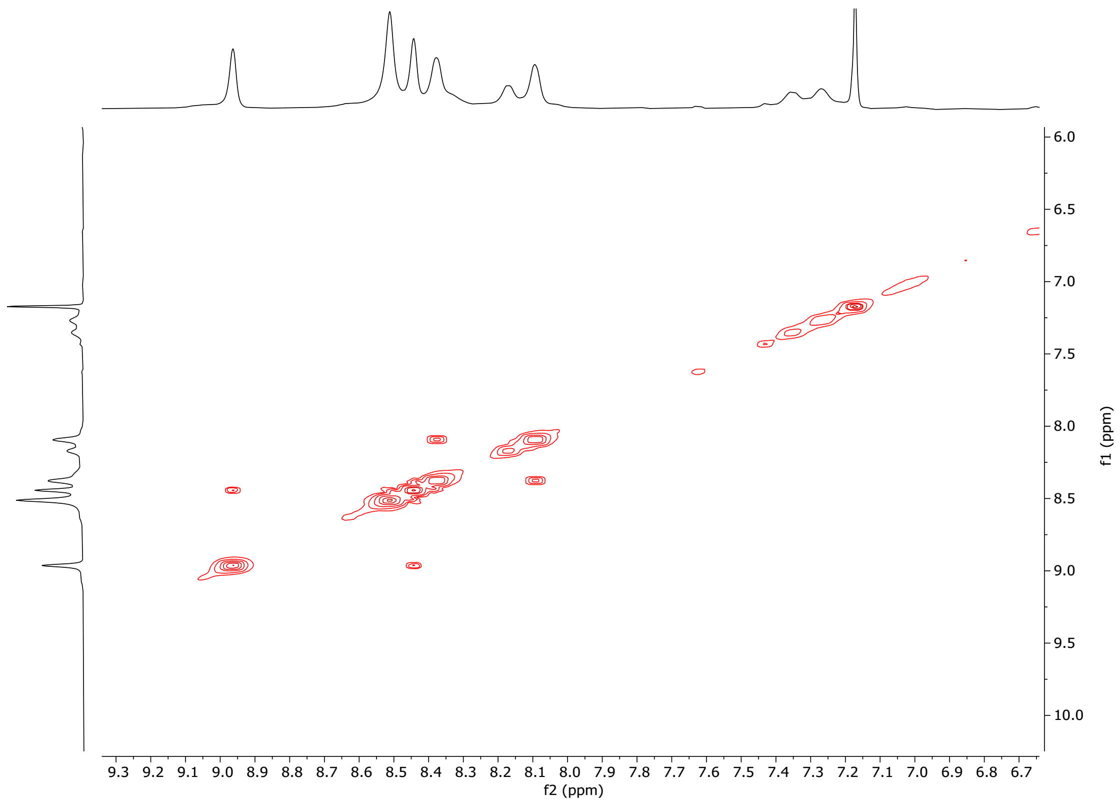

Figure S4. ^{1}H-^{1}H COSY 2D NMR spectrum of corrole **H_3LapCor** in $CDCl_3$.

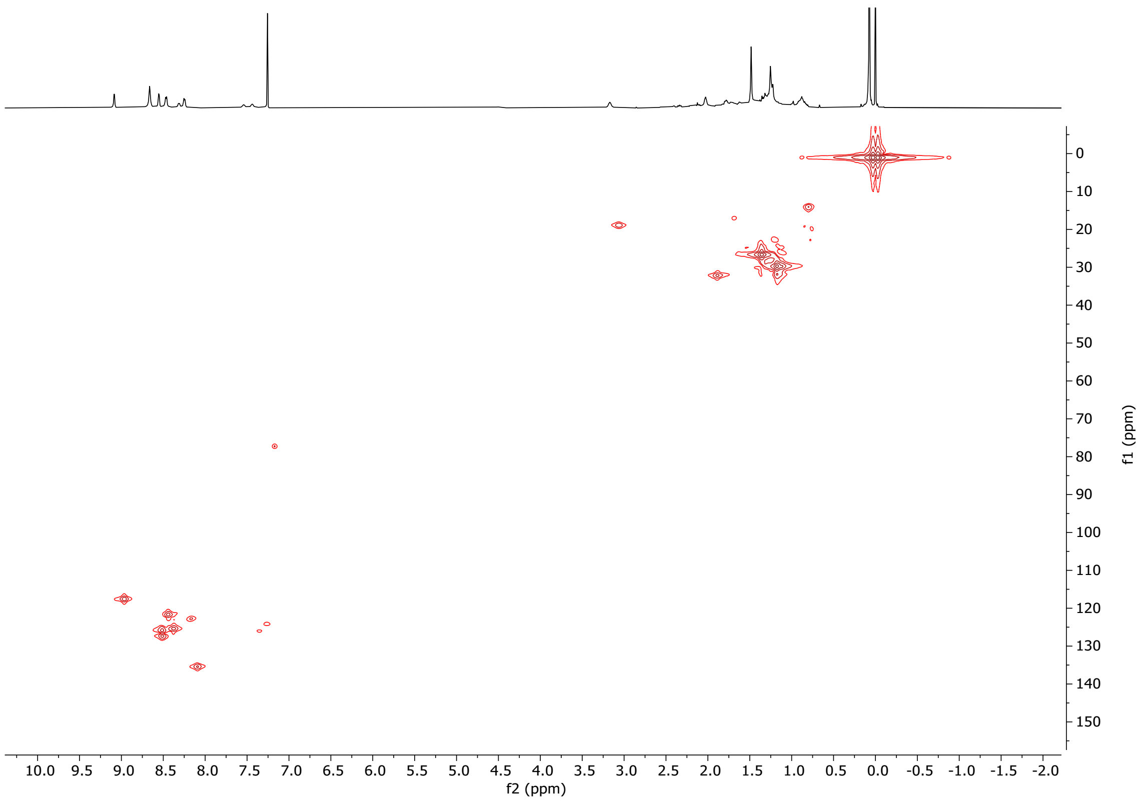

Figure S5. ^{1}H-^{13}C HMBC NMR spectrum of corrole **H_3LapCor** in $CDCl_3$.

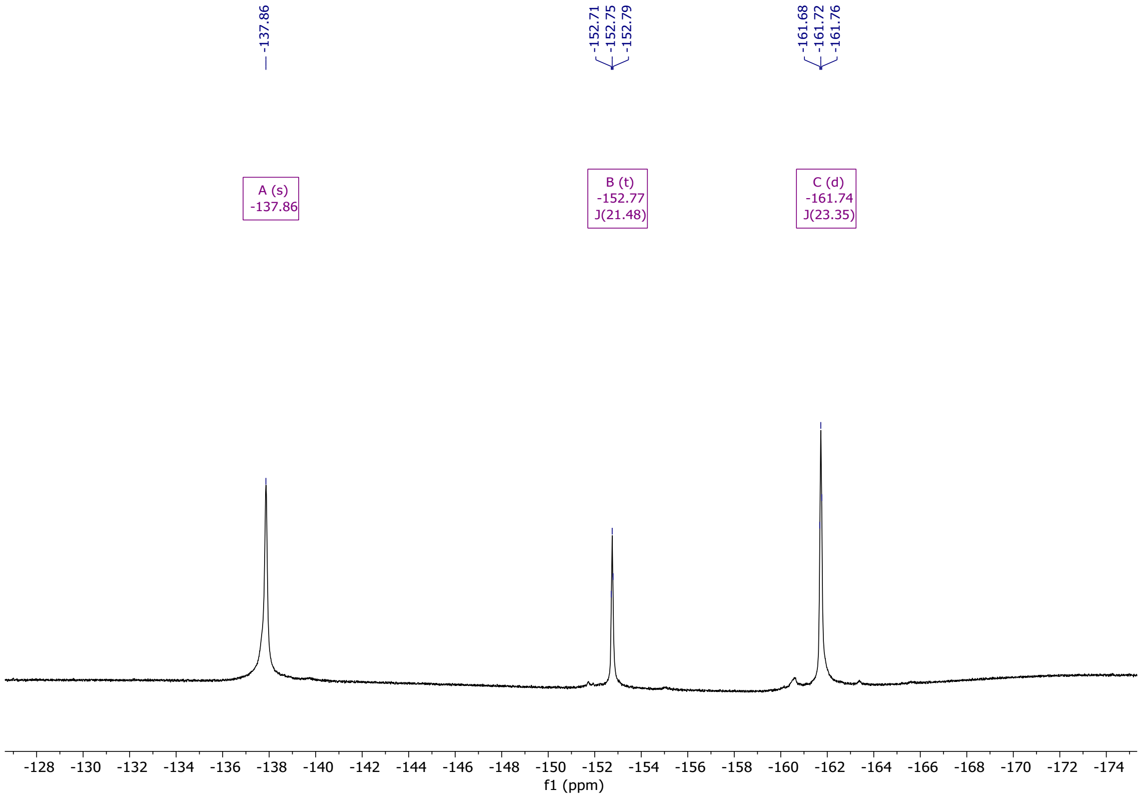

Figure S6. ^{19}F NMR spectrum (565 MHz) of corrole **H_3LapCor** in $CDCl_3$.

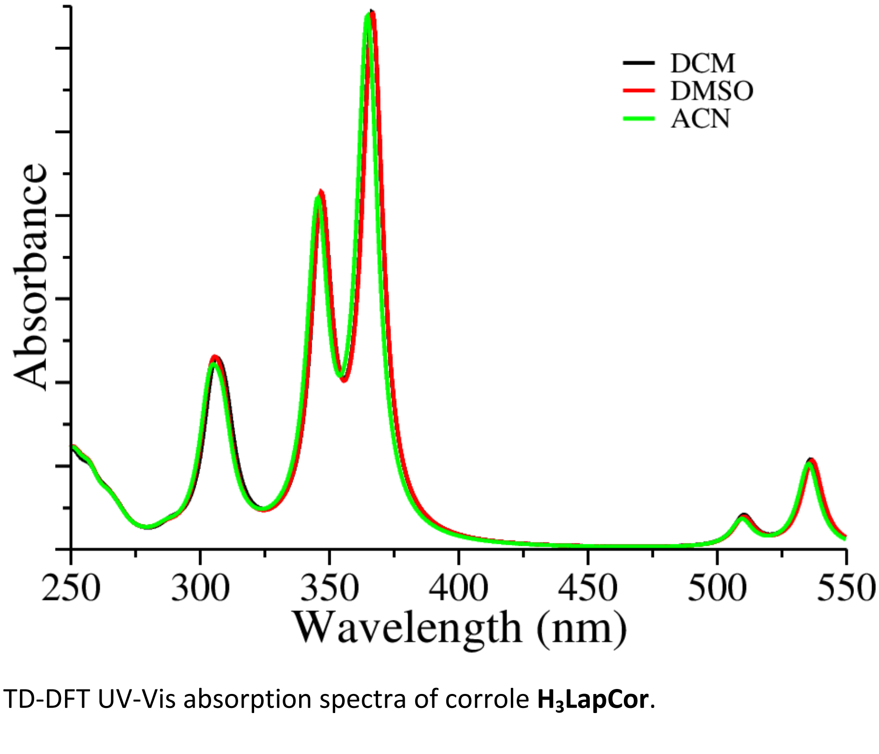

Figure S7. TD-DFT UV-Vis absorption spectra of corrole **H_3LapCor**.

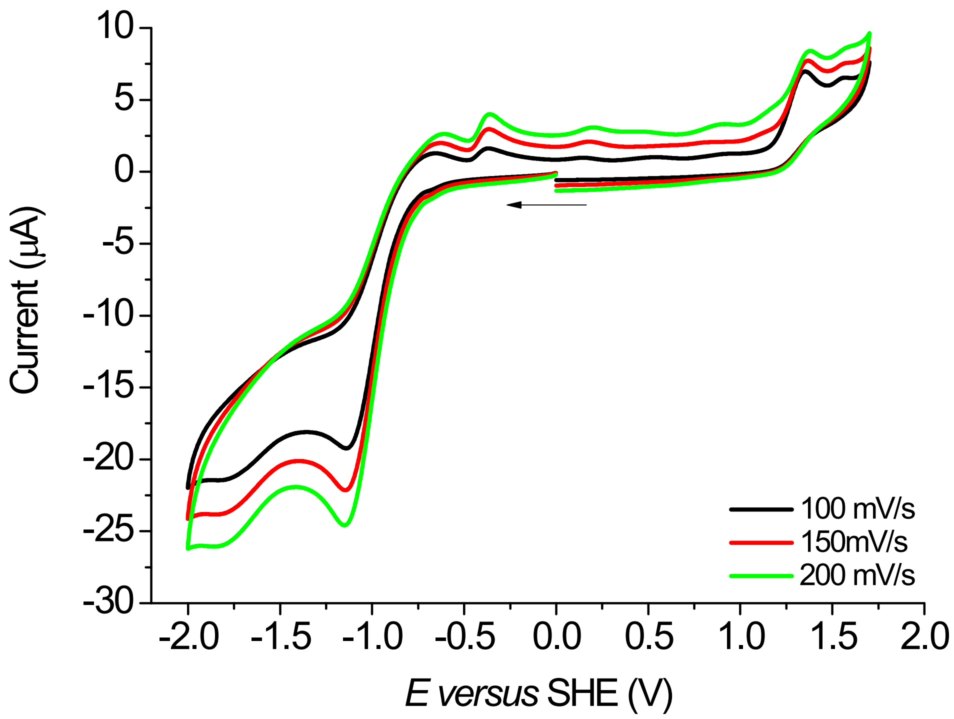

Figure S8. Cyclic voltametry of corrole **H_3LapCor** in dry DCM solution.

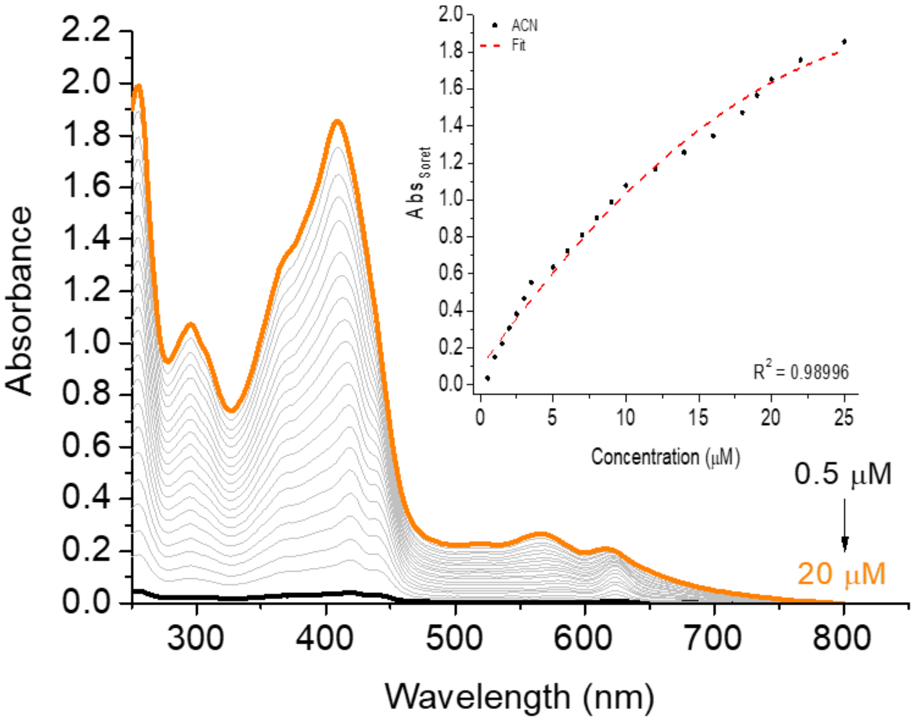

Figure S9. UV-Vis aggregation assays of corrole **H_3LapCor** in ACN solution, in the concentration variation from 0.5 to 20 μM. Inset: Abs_{Soret} versus concentration plot.

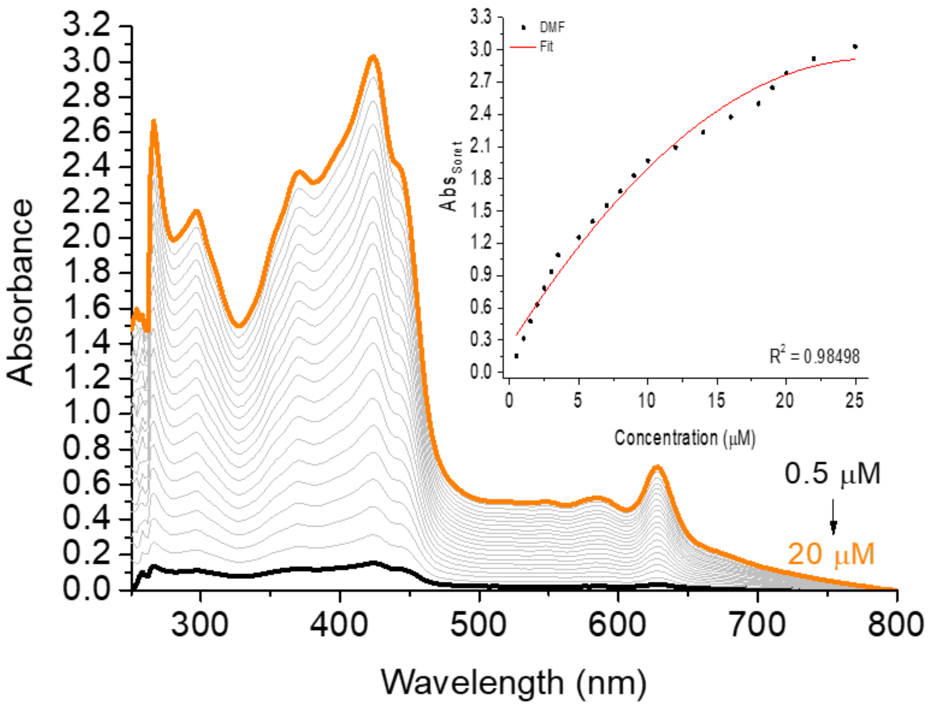

Figure S10. UV-Vis aggregation assays of corrole **H_3LapCor** in DMF solution, in the concentration variation from 0.5 to 20 µM. Inset: Abs_{Soret} versus concentration plot.

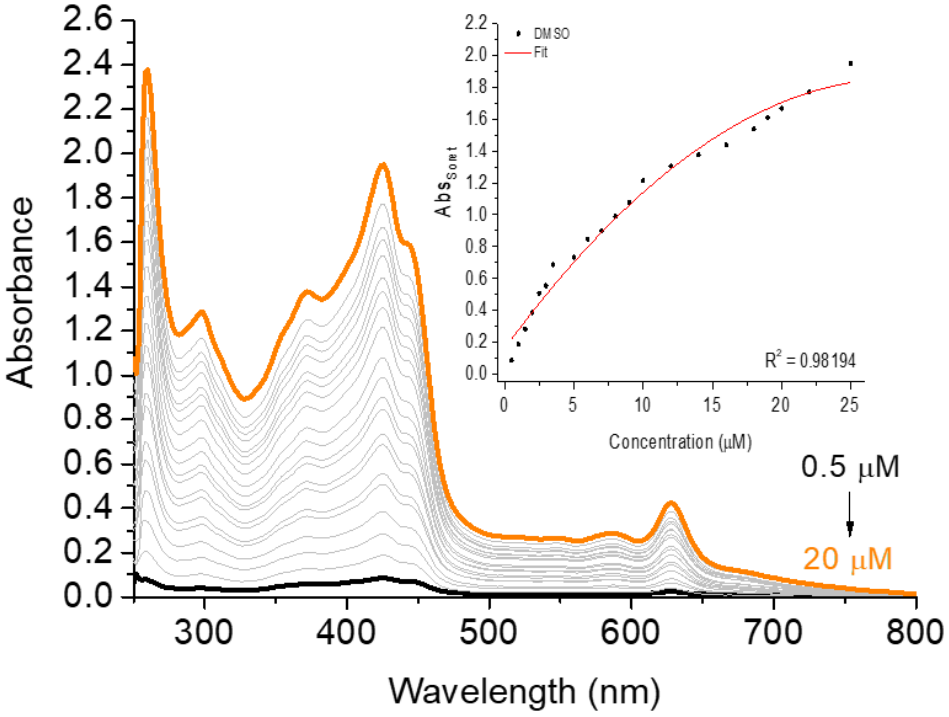

Figure S11. UV-Vis aggregation assays of corrole **H_3LapCor** in DMSO solution, in the concentration variation from 0.5 to 20 µM. Inset: Abs_{Soret} versus concentration plot.

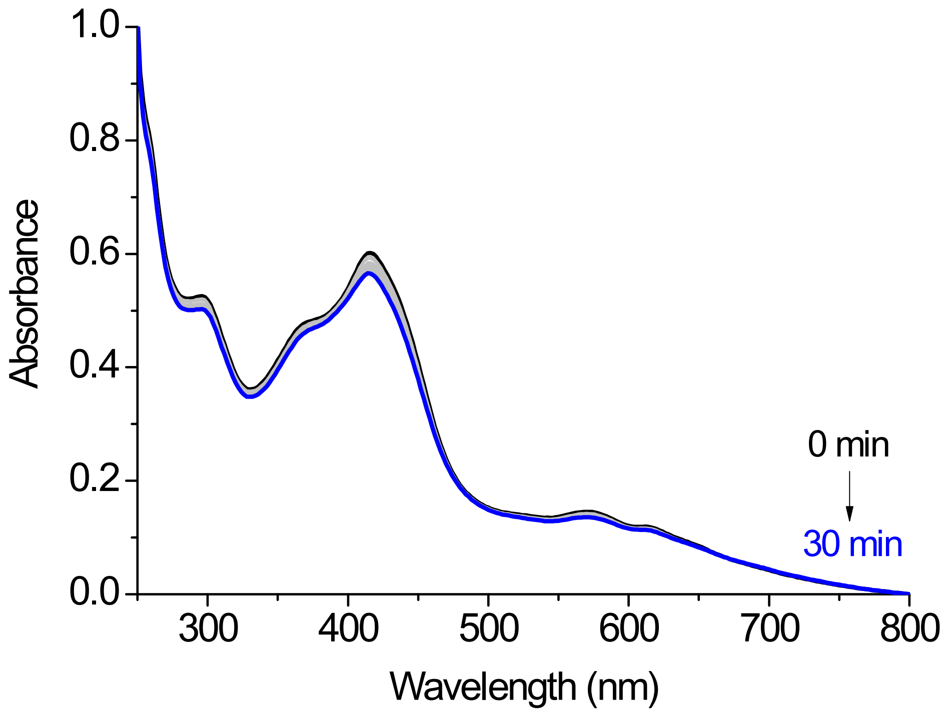

Figure S12. Photostability assays by UV-Vis analysis of corrole **H_3LapCor** in DMSO(5%)/Tris-HCl pH 7.4 buffer mixture solution, in white-light irradiation conditions, at period of 30 min.

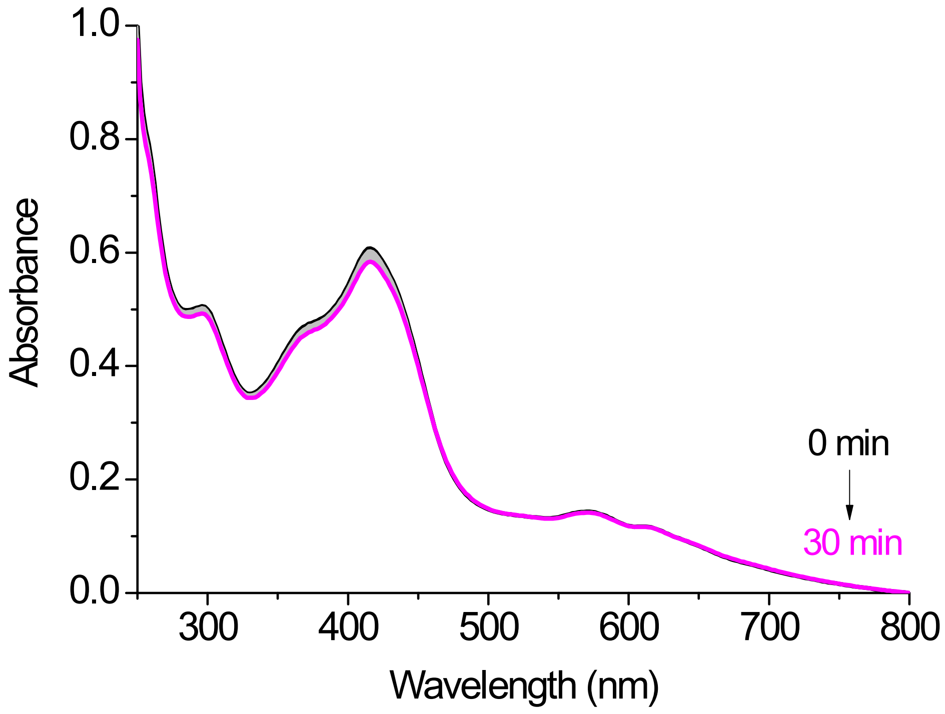

Figure S13. Photostability assays by UV-Vis analysis of corrole **H_3LapCor** in DMSO(5%)/Tris-HCl pH 7.4 buffer mixture solution, in red-light irradiation conditions, at period of 30 min.

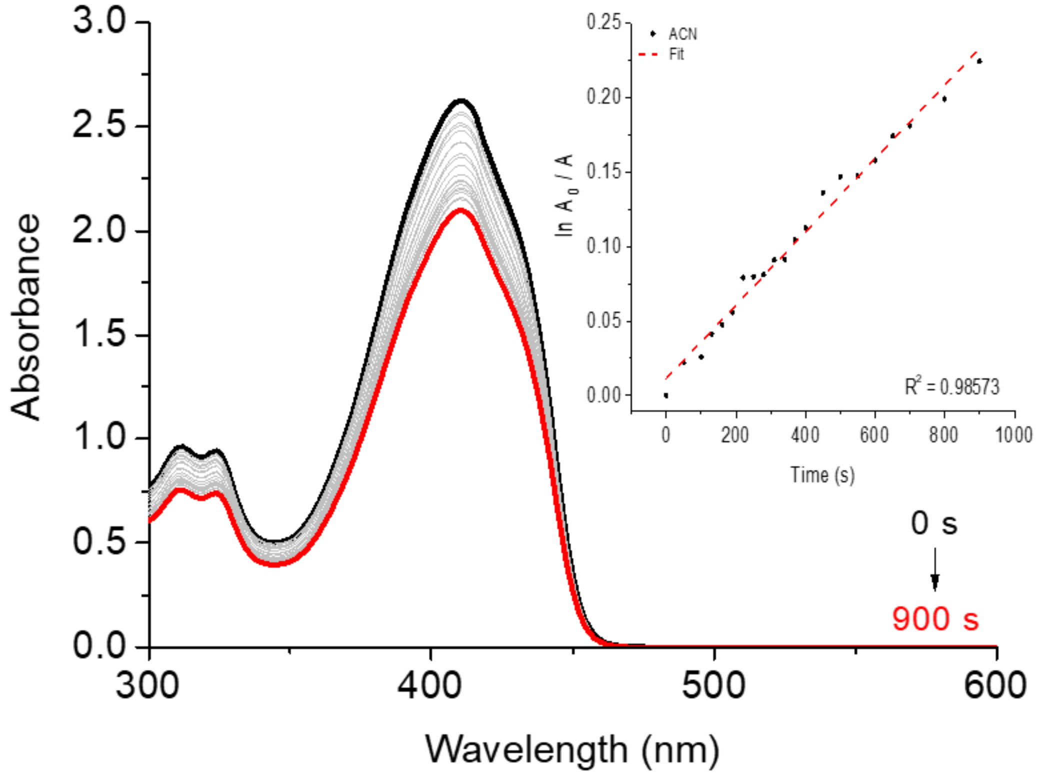

Figure S14. DPBF photo-oxidation experiment in ACN solution by irradiation with red-light LED source (660 nm; irradiance of 100 mW cm^{-2} and a total light dosage of 90 J cm^{-2}) in the presence of corrole **H_3LapCor**. The inset shows the first order kinetic profile.

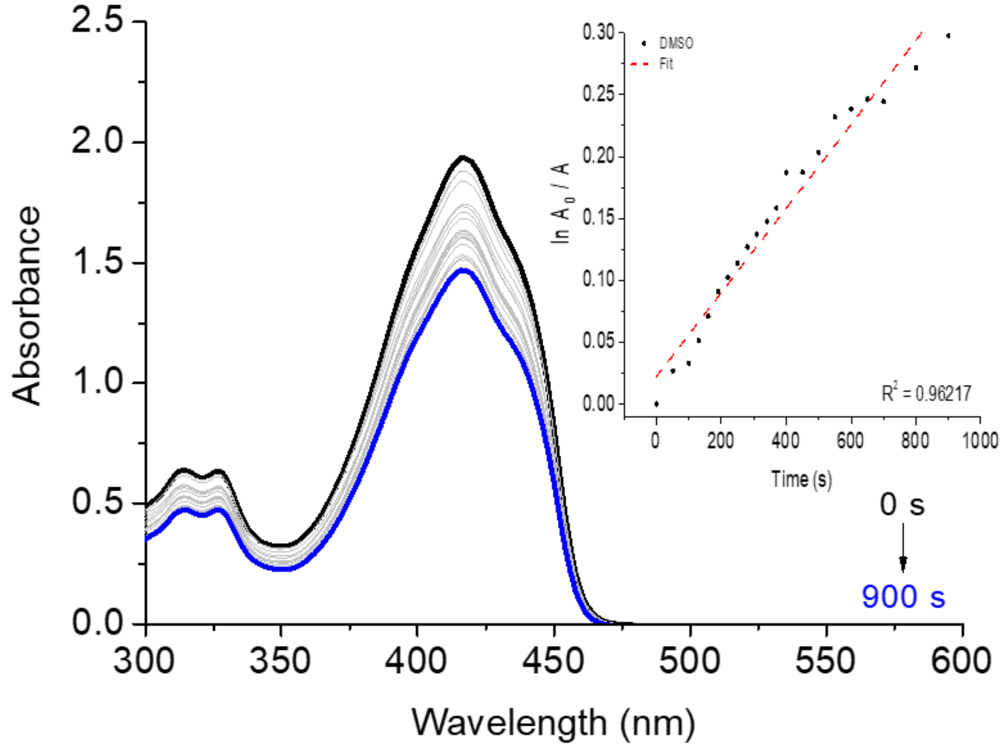

Figure S15. DPBF photo-oxidation experiment in DMSO solution by irradiation with red-light LED source (660 nm; irradiance of 100 mW cm^{-2} and a total light dosage of 90 J cm^{-2}) in the presence of corrole **H_3LapCor**. The inset shows the first order kinetic profile.

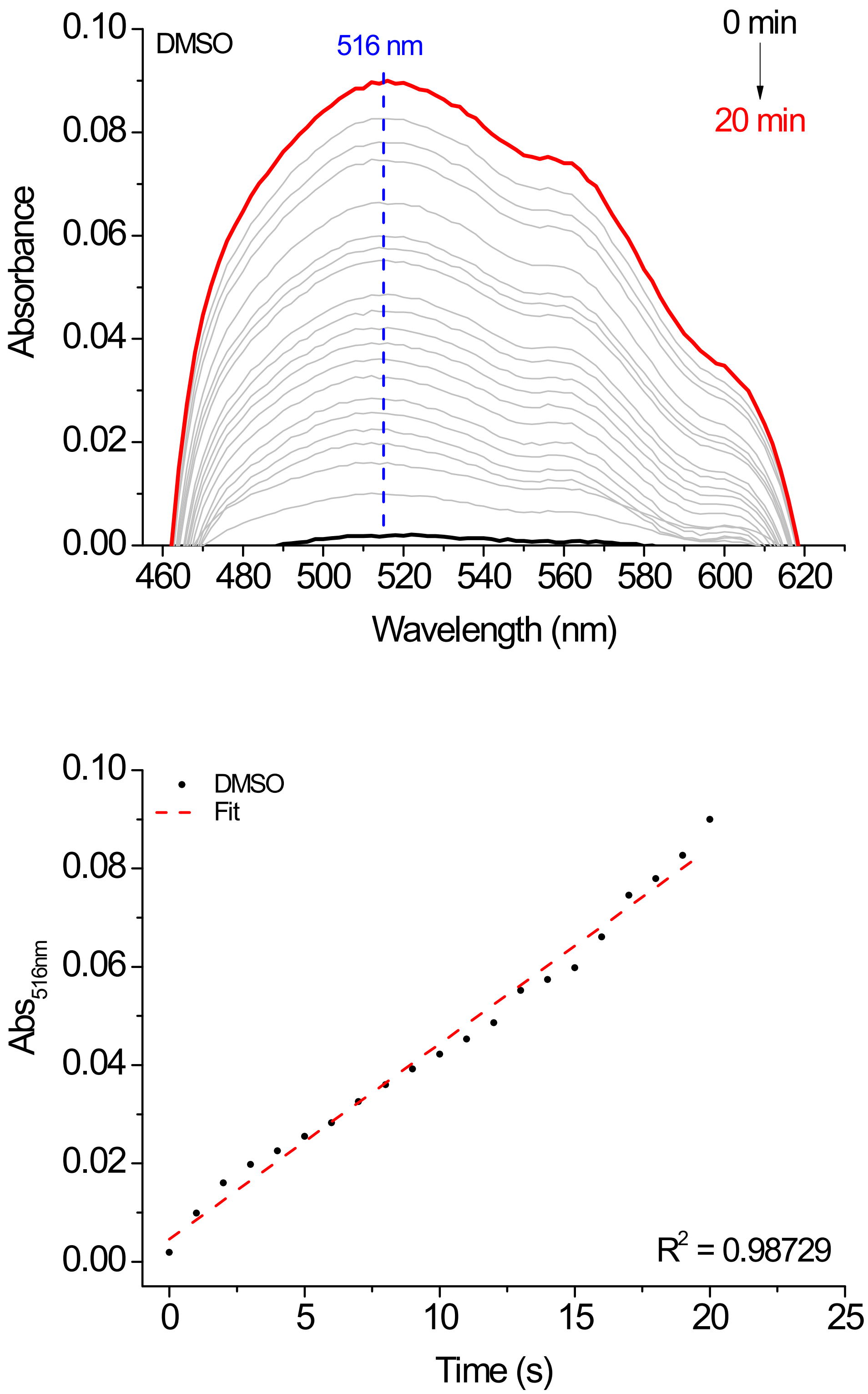

Figure S16. NBT reduction experiment in DMSO solution by irradiation with white-light LED source in the presence of corrole $\mathbf{H_3LapCor}$.

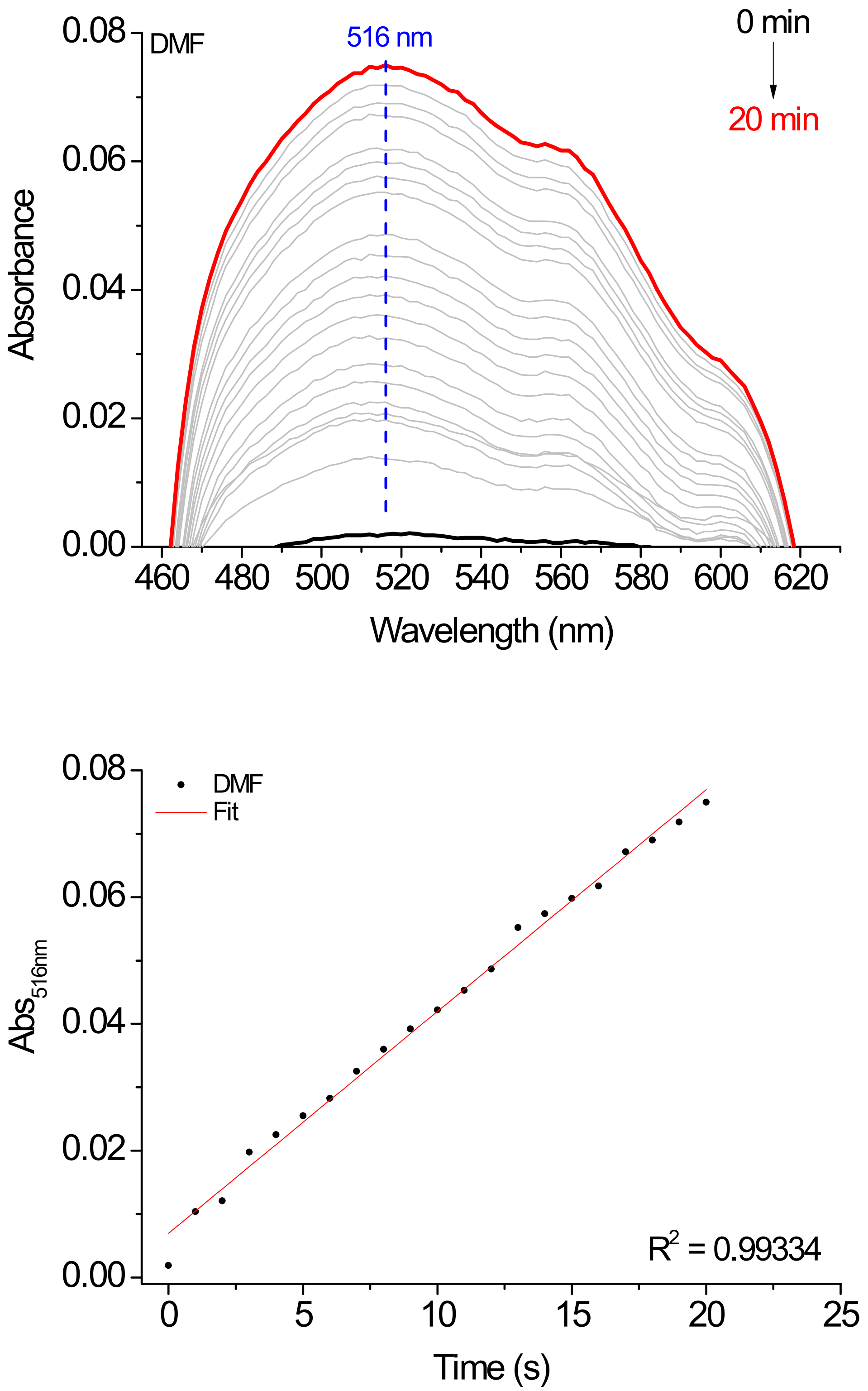

Figure S17. NBT reduction experiment in DMF solution by irradiation with white-light LED source in the presence of corrole **H_3LapCor**.

Table S1. Vertical transitions, Energy, oscillator strength (f), and Dipole Moment for the corrole-lapachone in three different solvent environments.

Environment	Transition	Energy eV(nm)	f	Dipole Moment
DCM	$S_0 \rightarrow S_1$	2.3124 (536.17)	0.3321	7.1398
	$S_0 \rightarrow S_2$	2.4297 (510.29)	0.1145	
	$S_0 \rightarrow S_3$	3.3820 (366.60)	1.9428	
	$S_0 \rightarrow S_4$	3.5742 (346.89)	1.2044	
DMSO	$S_0 \rightarrow S_1$	2.3100 (536.74)	0.3287	7.7482
	$S_0 \rightarrow S_2$	2.4294 (510.34)	0.1085	
	$S_0 \rightarrow S_3$	3.3812 (366.68)	1.9382	
	$S_0 \rightarrow S_4$	3.5751 (346.79)	1.2154	
ACN	$S_0 \rightarrow S_1$	2.3160 (535.35)	0.3287	7.6981
	$S_0 \rightarrow S_2$	2.4335 (509.49)	0.1085	
	$S_0 \rightarrow S_3$	3.3994 (364.73)	1.9382	
	$S_0 \rightarrow S_4$	3.5897 (345.39)	1.2154	
